# Modeling of the ComRS Signaling Pathway Reveals the Limiting Factors Controlling Competence in *Streptococcus thermophilus*

**DOI:** 10.3389/fmicb.2015.01413

**Published:** 2015-12-22

**Authors:** Laurie Haustenne, Georges Bastin, Pascal Hols, Laetitia Fontaine

**Affiliations:** ^1^Biochimie, Biophysique et Génétique des Microorganismes, Institut des Sciences de la Vie, Université catholique de LouvainLouvain-la-Neuve, Belgium; ^2^Center for Systems Engineering and Applied Mechanics, ICTEAM, Université catholique de LouvainLouvain-la-Neuve, Belgium

**Keywords:** timing device, feedback regulation, competence shut-off, ordinary differential equations, kinetics model, oscillation

## Abstract

In streptococci, entry in competence is dictated by ComX abundance. In *Streptococcus thermophilus*, production of ComX is transient and tightly regulated during growth: it is positively regulated by the cell-cell communication system ComRS during the activation phase and negatively regulated during the shut-off phase by unidentified late competence gene(s). Interestingly, most *S. thermophilus* strains are not or weakly transformable in permissive growth conditions (i.e., chemically defined medium, CDM), suggesting that some players of the ComRS regulatory pathway are limiting. Here, we combined mathematical modeling and experimental approaches to identify the components of the ComRS system which are critical for both dynamics and amplitude of ComX production in *S. thermophilus*. We built a deterministic, population-scaled model of the time-course regulation of specific ComX production in CDM growth conditions. Strains LMD-9 and LMG18311 were respectively selected as representative of highly and weakly transformable strains. Results from *in silico* simulations and *in vivo* luciferase activities show that ComR concentration is the main limiting factor for the level of *comX* expression and controls the kinetics of spontaneous competence induction in strain LMD-9. In addition, the model predicts that the poor transformability of strain LMG18311 results from a 10-fold lower *comR* expression level compared to strain LMD-9. In agreement, *comR* overexpression in both strains was shown to induce higher competence levels with deregulated kinetics patterns during growth. In conclusion, we propose that the level of ComR production is one important factor that could explain competence heterogeneity among *S. thermophilus* strains.

## Introduction

Genetic plasticity is at the core of the adaptation and evolution strategies in bacteria. Horizontal gene transfer (HGT) events allow individuals to rapidly acquire/lose phenotypic traits and have a major impact on bacterial evolution. Conjugation, transduction, and competence are the main HGT mechanisms, and define genetic exchange communities (Skippington and Ragan, 2011). Competence is defined as a transient physiological state that enables bacteria and archaea to take up exogenous naked DNA and to stably integrate it in their genome by homologous recombination (Johnsborg et al., 2007; Seitz and Blokesch, 2013). The transforming DNA can be passively or actively released from surrounding dead cells or siblings via a programmed cell lysis mechanism promoted by competent cells, respectively (Claverys et al., 2007; Berg et al., 2012; Wei and Havarstein, 2012; Borgeaud et al., 2015). Competence is increasingly viewed as a stress response that may increase adaptability and fitness in hostile conditions (Claverys et al., 2006; Perry et al., 2009; Charpentier et al., 2012; Dufour and Levesque, 2013).

DNA transformation is an energy-consuming process with possible deleterious effects on the population fitness due, for instance, to the acquisition of deleterious mutations and a slower growth rate of competent cells (Haijema et al., 2001; Johnsen et al., 2009; Moradigaravand and Engelstadter, 2013). To optimize the benefits vs. cost of natural transformation, bacteria have implemented complex and interconnected regulatory systems to turn on competence only when particular environmental and physiological parameters reach a critical point (for recent reviews, see Seitz and Blokesch, 2013; Johnston et al., 2014; Fontaine et al., 2015). For instance, streptococci use cell-cell communication systems to synchronize competence induction between individuals, allowing them to behave such as a multicellular organism. This coordination relies on the production and sensing of peptidic competence pheromones. Their production and secretion is generally initiated in response to specific environmental stresses (Claverys et al., 2006; Fontaine et al., 2015). At a critical extracellular concentration, competence pheromones activate the production of the master competence regulator in streptococci, ComX. ComX is a sigma factor (σ^X^) that associates to the RNA polymerase to redirect transcription toward genes required for DNA transport and processing (also named late *com* genes). Competence pheromones also activate a positive feedback loop on their production that is thought to be necessary to coordinate and maintain competence induction in the population. Two main communication systems have been reported to directly control *comX* expression. The ComCDE three-component system is prevalent in species belonging to the mitis and anginosus taxonomic groups such as *Streptococcus pneumoniae* and *Streptococcus anginosus*, respectively. This system will not be extensively detailed here (For a recent review, see Fontaine et al., 2015). Briefly, the secreted competence pheromones (encoded by *comC*) are detected in the medium by the histidine kinase ComD, which transmits the information by phosphorelay to ComE. ComE~P then activates the transcription of *comX* and *comCDE*, thereby activating a positive feedback loop. The second system, named ComRS, is present in species belonging to the salivarius, mutans, pyogenic, suis, and bovis groups. Together with *comX*, the encoding genes of both ComCDE and ComRS communication systems belong to the so-called early *com* genes (Fontaine et al., 2015).

**Figure 1:**
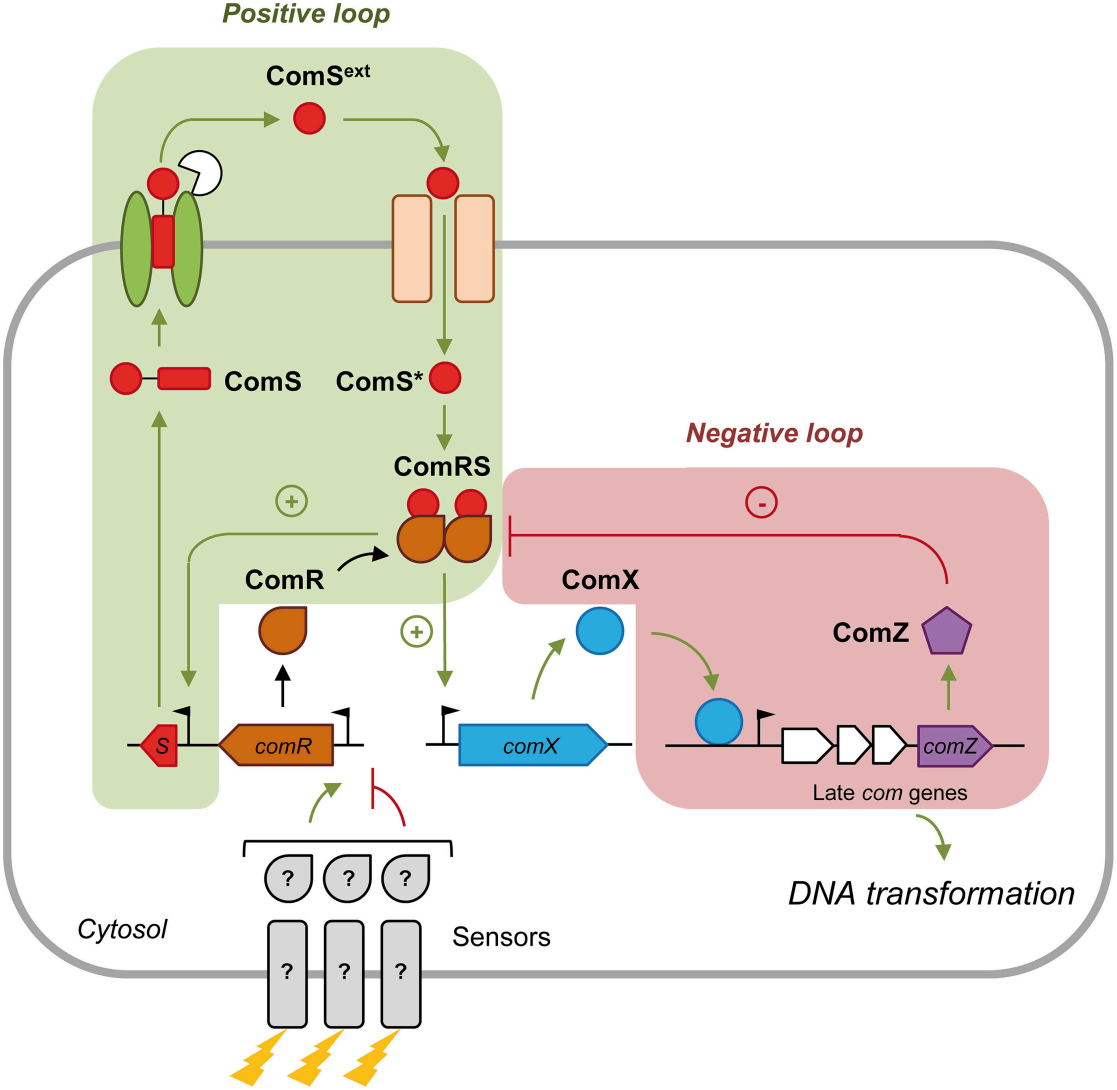
**Regulation of ***comX*** expression by the ComRS signal transduction system in streptococci**. Competence development is initiated by the production of the precursor of the competence pheromone ComS. ComS is then exported by an unknown transporter and matured by species-specific protease(s). At a critical concentration, the mature competence peptide ComS^ext^ (also named XIP) is sensed by a specific oligopeptide-binding protein OppA/AmiA and reimported through the Opp/Ami transporter. In the cytoplasm, the intracellular mature peptide ComS^*^ interacts with the transcriptional regulator ComR. The resulting dimeric ComRS complex activates the transcription of early competence genes (green arrows) including *comS* (plus sign; positive feedback loop) and *comX* by binding to their promoter. Expression of *comR* is not activated by ComRS and is thought to be regulated by other regulators/sensors (?) in response to specific environmental cues (represented by lightning). ComX or σ^X^ induces transcription of late competence genes necessary for DNA transformation. Competence shut-off (red lines) in *S. thermophilus* involves at least one or more late gene product(s), named here ComZ. ComZ is thought to interact with and inactivate the ComRS complex (minus sign; negative feedback loop). The names of modeled peptidic/proteic species in system S1 are in black bold.

Growth in a chemically defined medium (CDM) devoid of complex oligopeptides seems to be optimal for ComRS activation (Mashburn-Warren et al., 2010; Desai et al., 2012; Son et al., 2012; Wenderska et al., 2012; Gardan et al., 2013; Morrison et al., 2013; Zaccaria et al., 2014). The best studied models are *Streptococcus mutans* (mutans group) and *Streptococcus thermophilus* (salivarius group) (Figure 1). In CDM, the ComS precursor is produced and secreted by as yet undiscovered transporter(s). During or after its secretion, ComS is matured by species-specific protease(s) (Khan et al., 2012; Gardan et al., 2013) to release the C-terminal pheromone domain named XIP (for σ^X^ inducing peptide; ComS^ext^ in Figure 1) (minimum active size is between 7 and 11 aa, see Fontaine et al., 2015). At a critical extracellular concentration, XIP peptides are re-imported into the cytoplasm through the Opp/Ami transporter (Gardan et al., 2009, 2013; Mashburn-Warren et al., 2010; Fontaine et al., 2010a). XIP then interacts with the transcriptional activator ComR and stimulates the binding of the ComR-XIP complex as a dimer to the ComR-box located in the promoter of *comX* and *comS*, thereby promoting their transcription. The ComR-XIP complex induces a positive feedback loop on ComS production, but not on ComR. In agreement, the *comR* promoter is devoid of any ComR-box (Fontaine et al., 2013). The control of ComR production is believed to be the target of distal regulatory systems in response to environmental cues (Mashburn-Warren et al., 2010; Fontaine et al., 2013, 2015). In *S. mutans*, distal *comR* regulators are the BsrRM-HdrRM relay in response to cell density (Okinaga et al., 2010; Xie et al., 2010) and the ScnRK two-component system involved in oxidative stress resistance (Kim et al., 2013).

The shut-off mechanism of competence in ComRS-containing streptococci has been poorly investigated. In *S. thermophilus*, deletion of *comX* increases the level and duration of early *com* genes expression (e.g., *comS*) (Boutry et al., 2013), similarly to the case of *S. pneumoniae* (Mirouze et al., 2013; Weng et al., 2013). This suggests that some ComX-regulated genes are involved in this process. In *S. mutans*, active proteolysis of σ^X^ by the MecA-ClpCP machinery plays an important role in the kinetics of competence shut-off (Tian et al., 2013).

Kinetics of *comX* induction in CDM differs remarkably between species and strains (Desai et al., 2012; Gardan et al., 2013; Morrison et al., 2013; Zaccaria et al., 2014) and is greatly influenced by growth parameters such as pH (Guo et al., 2014; Son et al., 2015). For instance, the ComRS system of *S. thermophilus* is viewed as a timing device, rather than a true quorum-sensing system. Indeed, spontaneous expression of early *com* genes starts to increase at the early log phase, whatever the size of inocula, and lasts for about 1 h. In addition, induction of competence by XIP in *S. thermophilus* is less efficient in the lag and stationary phases (Gardan et al., 2013). This suggests that regulatory components need to be synthesized, or alternatively, degraded for optimal ComRS signaling (Fontaine et al., 2015).

As the ComRS system was recently discovered, many questions about its regulatory cascade still remain open. For instance, it is not clear how environmental parameters are integrated to fine-tune the activity of the ComRS complex. In this context, few ComRS-containing strains turn out to be spontaneously transformable in CDM, but most are responsive to artificial induction (Gardan et al., 2009; Fontaine et al., 2010a,b; Desai et al., 2012; Son et al., 2012; Morrison et al., 2013; Zaccaria et al., 2014). This suggests that some important steps of the regulatory cascade may either be limiting or missing in these growth conditions, which are far from those encountered in natural habitats.

The aim of this study is to deeply re-examine the ComRS regulatory cascade and identify the critical components for ComX production in species belonging to the salivarius group, using a combination of mathematical modeling and experimental molecular approaches. Based on the previous knowledge acquired on the ComRS regulatory cascade, *S. thermophilus* was chosen as a model bacterium. Strains LMD-9 and LMG18311 were respectively selected as representative of highly and weakly transformable strains (Fontaine et al., 2010b). A dynamic and mechanistic model of the ComRS system of strain LMD-9, partially calibrated by experimental data, was first built. This model, that integrates growth parameters, was then used to study qualitative and quantitative aspects (topology and dynamics) of the ComRS system in order to identify the regulatory determinants of competence development in *S. thermophilus*.

## Materials and methods

### Experimental procedures

#### Bacterial strains, plasmids, and growth conditions

The bacterial strains and plasmids used in the present study are listed in Supplementary Table S1. Plasmids derived from pGICB004 were specifically constructed in *Escherichia coli* EC1000. *E. coli* was grown in LB medium with shaking at 37°C (Sambrook et al., 1989). *S. thermophilus* was grown at 37°C in M17 broth (Difco Laboratories Inc., Detroit, MI) or in CDM, as described by Letort and Juillard (2001). All synthetic media contain 1% (wt vol^−1^) lactose (M17L and CDML broth, respectively). When required, spectinomycin (200 μg ml^−1^ for *E. coli* and *S. thermophilus*), erythromycin (250 μg ml^−1^ for *E. coli*, 2.5 μg ml^−1^ for *S. thermophilus*) or chloramphenicol (20 μg ml^−1^ for *E. coli*, 5 μg ml^−1^ for *S. thermophilus*) was added to the media. Solid agar plates were prepared by adding 2% (wt vol^−1^) agar to the medium. Solid plates inoculated with *S. thermophilus* cells are incubated anaerobically (BBL GasPak systems, Becton Dickinson, Franklin lakes, NJ) at 37°C.

#### Detection of absorbance and luminescence

Small volumes (300 μl) of culture samples (OD_600_ of 0.05) were incubated in the wells of a sterile covered white microplate with a transparent bottom (Greiner, Alphen a/d Rijn, The Netherlands). Growth (OD_600_) and luciferase (Lux) activity (expressed in relative light unit; RLU) were monitored at 10-min intervals during 5 h in a Varioskan Flash multi-mode reader (ThermoFisher Scientific, Zellic, Belgium) as previously described (Fontaine et al., 2010a, 2013). In the supplementation experiments, different concentrations of synthetic forms of ComS_17-24_ (LPYFAGCL) (purity >95%) supplied by Peptide 2.0 (Chantilly, VA) were added to the 300 μl-culture samples after 1 h of growth at 37°C.

#### Natural transformation experiments

Experiments were performed as previously described (Fontaine et al., 2010a,b, 2013). The DNA, either pGIUD0855cat (1 μg), pGILFspec::P_*comR*_ (1 μg) or purified overlap PCR products (25 ng), were added to 300 μl culture samples. The transformation frequency was calculated as the number of antibiotic-resistant CFUs per ml divided by the total number of viable CFUs per ml. Antibiotic-resistant CFUs were selected on chloramphenicol in case the transforming DNA is pGIUD0855cat or a P32*-cat*-encompassing PCR product, or on spectinomycin when the transforming DNA encodes a P_spec_-*spec* cassette (plasmid pGILFspec::P_*comR*_). Integration of the antibiotic resistance cassette at the right location in the chromosome of transformants was checked by PCR (primer pairs used are listed in Supplementary Table S2).

#### DNA techniques and electrotransformation

General molecular biology techniques were performed according to the instructions given by Sambrook et al. (1989). *S. thermophilus* LMD-9 and *S. thermophilus* LMG18311 chromosomal DNA was prepared as described by Ferain et al. (1996). Electrotransformation of *E. coli* was performed as described by Dower et al. (1988). Electrocompetent *S. thermophilus* cells were prepared as previously described (Blomqvist et al., 2006). After transformation with 1 μg of plasmid DNA, cells were immediately resuspended in 1 ml M17L and incubated anaerobically during 6 h at 37°C (pMG36e derivatives) (van de Guchte et al., 1989) or 29°C (pGICB001) (Law et al., 1995). PCRs were performed with Phusion high-fidelity DNA polymerase (Finnzymes, Espoo, Finland) in a GeneAmp PCR system 2400 (Applied Biosystems, Foster City, CA). The primers used in this study were purchased from Eurogentec (Seraing, Belgium) and are listed in Supplementary Table S2.

#### Deletion of DprA by natural transformation

The DprA^−^ derivatives of LMD-9 reporter strains were constructed by exchanging the *dprA* open reading frame (ORF) (sequence between the start and stop codons) with the chloramphenicol resistance cassette *lox66*-P32-*cat-lox71*, as previously described (Fontaine et al., 2010b). Primers used are listed in Supplementary Table S2.

#### Construction of reporter strains

Luminescent LMG18311 reporter strains CB009 and LF146 were constructed by replacing part of the *blp* locus (from *blpU* to *blpX*) by the transcriptional fusion P_*comX*_-*luxAB* and P_*comR*_-*luxAB-spec*, respectively. This was achieved by transforming pGICB004 derivatives, respectively pGICB001 and pGILF::P_*comR*_, which carries *blp* recombination fragments, as previously described (Fontaine et al., 2010a; Fleuchot et al., 2011). Plasmid pGILFspec::P_*comR*_ was obtained in two steps. First, the spectinomycin resistance cassette was cloned between *luxAB* and the *blp* recombination sequence of pGICB004 in order to easily select the desired transformants on spectinomycin. To this end, P_spec_-*spec* was amplified by PCR from plasmid pR412 with primer pairs LoxSpec-SmaI-F/LoxSpec-Pvu-R, digested by *Sma*I and *Pvu*II and cloned in pGICB004 linearized by *Sma*I, yielding pGILFspec. Second, a PCR product corresponding to the P_*comR*_ sequence from LMG18311 and digested by *EcoR*I and *Spe*I was cloned in similarly digested plasmid pGILFspec (see Supplementary Tables S1, S2).

#### EMSA experiments

The double-stranded DNA fragment Cy3-P_*comS*_ used in the EMSA experiments (approximately 200 bp) was amplified by PCR from *S. thermophilus* LMD-9 (Supplementary Table S2). The 5′ end of the forward primer used was coupled to the Alexa 555 fluorophore. ComR-Strep from *S. thermophilus* LMD-9 was purified as previously described (Fontaine et al., 2013). Typically, a gel shift reaction (20 μl) was performed in a binding buffer (20 mM Tris-HCl pH 8.0, 150 mM NaCl, 1 mM EDTA, 1 mM DTT, 10% glycerol, 1 mg ml^−1^ BSA) and contained 150 ng labeled probe and 4 μM ComR-Strep proteins. When necessary, 0.2 or 2 μM of ComS_17-24_ peptides are added. The reaction is incubated at 37°C for 10 min prior to loading of the samples on a native TBE 5% gel. The gel is next subjected to 80 V for approximately 1 h in TBE buffer. DNA complexes were detected by fluorescence on the Ettan DIGE Imager with bandpass excitation filters (nm) of 540/25 (Cy3) and bandpass emission filters of 595/25 (Cy3) (GE Healthcare, Waukesha, WI).

### Mathematical modeling

Our model describes the mean behavior of a competent cell in a homogenous *S. thermophilus* LMD-9 population grown in CDML conditions. The model focuses on the timing device function of the ComRS system, solely. In this context, ComS exportation/maturation and importation are modeled to create a time delay in the signaling cascade. The choice of not modeling the cell-cell communication aspect of ComS in *S. thermophilus* is here justified by experimental evidences that (i) secreted mature ComS peptides (XIP) do not freely diffuse in CDML medium and do rather remain associated to the surface of secreting cells (Gardan et al., 2013) and that (ii) the competence cycle (on- and off-steps) takes place at low cell densities (at least in CDML growth conditions) (Gardan et al., 2009, 2013; Fontaine et al., 2010a).

System S1 is composed of seven deterministic ordinary differential equations (ODE) describing the time-course evolution of the seven proteic/peptidic species involved in the (transcriptional) control of ComX production: precursor peptides ComS, secreted peptides ComS^ext^, intracellular mature peptides ComS^*^, ComR, active complexes ComRS, ComX, and ComZ. These variables are expressed in number of molecules per cell (Table 1). In addition, an equation representing the time-evolution of the cell density *X*(*t*) is added (Equation 1, expressed in number of cells per ml), since the development of competence is tightly coupled to the growth process. ComZ is a hypothetical actor that represents the protein(s) of the late phase responsible for competence shut-off. The description of variables and parameters of all equations is presented in Tables 1, 2, respectively.

**Table 1:** **Description of variables in system S1**.

**Variable**	**Description**	Unit^*^
*X*(*t*)	Cellular density	cell ml^−1^
*ComS*(*t*)	Concentration of precursor peptide ComS (24 aa)	mol. cell^−1^
*ComS*^*ext*^(*t*)	Concentration of external ComS peptides (mature pheromone)	mol. cell^−1^
ComS^*^(*t*)	Concentration of reimported mature ComS	mol. cell^−1^
*ComR*(*t*)	Concentration of ComR regulator	mol. cell^−1^
*ComRS*(*t*)	Concentration of active ComRS complex	mol. cell^−1^
*ComX*(*t*)	Concentration of alternative sigma factor ComX	mol. cell^−1^
*ComZ*(*t*)	Concentration of putative competence repressor ComZ	mol. cell^−1^

* *mol., molecules*.

**Table 2:** **Description and numerical values of parameters in system S1**.

**Symbol**	**Description**	**Cell-level unit**	Standard unit^*^
*r_*out*_*	Exportation rate constant	1 min^−1^	1 min^−1^
*r_*in*_*	Importation rate constant	0.01 min^−1^	0.01 min^−1^
*n*	Hill coefficient (Son et al., 2012)	2.5 (adim.)	2.5 (adim.)
*c_*RS*_*	Complex formation rate constant	0.1 mol.^−4^ cell^4^ min^−1^	1.2 × 10^−3^ nM^−4^ min^−1^
*V${}_{max}^{Z}$*	Maximal activation rate of ComZ by ComX	1 mol. cell^−1^ min^−1^	3 nM min^−1^
*n_*Z*_*	Hill coefficient	3 (adim.)	3 (adim.)
*K${}_{m}^{Z}$*	Required concentration of ComX for half-maximum synthesis rate of ComZ	15 mol. cell^−1^	45 nM
*V${}_{max}^{RSZ}$*	Maximal inactivation rate of ComRS by ComZ	0.04 mol. cell^−1^ min^−1^	0.12 nM min^−1^
*K${}_{m}^{RSZ}$*	Required concentration of ComRSComZ for half-maximum inactivation rate of ComRS	0.5 mol.^2^ cell^−2^	4.5 nM^2^
*d_*Sext*_*	Degradation rate constant of ComS^ext^	0.07 min^−1^	0.07 min^−1^
*d*_S_*__	Degradation rate constant of ComS^*^	0.07 min^−1^	0.07 min^−1^
*d_*R*_*	Degradation rate constant of ComR	0.01 min^−1^	0.01 min^−1^
*d_*RS*_*	Degradation rate constant of ComRS	0.01 min^−1^	0.01 min^−1^
*d_*X*_*	Degradation rate constant of ComX (Karlsson et al., 2007)	0.2 min^−1^	0.2 min^−1^
*d_*Z*_*	Degradation rate constant of ComZ	0.01 min^−1^	0.01 min^−1^
*V${}_{max}^{S}$*	Maximal activation rate of ComS by ComRS	4 mol. cell^−1^ min^−1^	12 nM min^−1^
*K${}_{m}^{S}$*	Required concentration of ComRS for half-maximum synthesis rate of ComS	0.5 mol. cell^−1^	1.5 nM
*A*	Amplitude of the gaussian activation of ComR	27 mol. cell^−1^	81 nM
*s*	Standard deviation of the activation of ComR	35 min	35 min
*m*	Center of the gaussian activation of ComR	90 min	90 min
*V${}_{max}^{X}$*	Maximal activation rate of ComX by ComRS	5.3 mol. cell^−1^ min^−1^	15.9 nM min^−1^
*K${}_{m}^{X}$*	Required concentration of ComRS for half-maximum synthesis rate of ComX	1 mol. cell^−1^	3 nM

* *Units of the systems are converted in standard units as follows: 1 molecule per cell corresponds to a molar concentration of 3 nM, assuming that a S. thermophilus cell is spherical with a diameter of 1 μm, which corresponds to a volume of 0.5 fl. mol., molecules; adim., adimensional*.

According to the structure of the regulatory network illustrated in Figure 1, the production of some variables (e.g., ComR, ComS, ComX) is controlled by a basal term *b*(*t*) and activation and/or repression terms. The time-course evolution of all variables is also negatively affected by a degradation term, which depends on a specific degradation rate *d* (we assume first-order degradation kinetics), and a dilution term which accounts for cell division and is thus directly related to the time-dependent growth rate μ(*t*).

#### ODE Equations of system S1

(1) $$\begin{array}{l} \frac{dX\left(t\right)}{dt}=\mu \left(t\right)X\left(t\right) \end{array}$$

Equation (1) models the growth kinetics of LMD-9 cells in CDML conditions. It allows to model competence development during the different growth phases (lag, exponential, and stationary). The time-varying cell density *X*(*t*) (expressed in cell ml^−1^) was computed from a mean experimental growth curve. To convert experimental OD_600_ units in number of cells per ml, we assumed that a OD_600_ unit of 1 = 5 × 10^8^ cells ml^−1^. This conversion was deduced from a plating and CFU (colony forming unit) counting experiment (data not shown). The varying growth rate μ(*t*) (expressed in min^−1^) was then computed from *X*(*t*) according to Equation (1) (see Supplementary Figure S1A)

(2) $$\begin{array}{l} \frac{dComS\left(t\right)}{dt}=b_{s}\left(t\right)+\underset{\text{activation by ComRS}}{\underset{︸}{V_{max}^{S}\left(\frac{ComRS\left(t\right)}{K_{m}^{S}+ComRS\left(t\right)}\right)}}-\underset{\text{exportation}}{\underset{︸}{r_{out}ComS\left(t\right)}}\\ -\;\underset{\text{growth}}{\underset{︸}{\mu \left(t\right)ComS\left(t\right)}} \end{array}$$

Equation (2) describes how the specific number (i.e., per cell) of cytoplasmic precursor ComS peptides (Figure 1) varies with time. ComS is produced in the cell at a non-constant basal rate *b*_*S*_(*t*), and its production is activated by the ComRS complex according to a Michaelis-Menten function, as modeled in Son et al. (2012). This activation term represents the positive feedback loop which activates and maintains ComS production. The term “exportation” on the right side represents the secretion of ComS in the extracellular medium. The latter is modeled as a non-saturating step: it depends on a constant exportation rate *r*_*out*_ and increases directly with *ComS*(*t*) concentration. ComS maturation was also assumed to be concomitant with exportation. The last term models ComS dilution due to cell division. The degradation rate of the cytoplasmic precursor ComS was assumed to be null, reflecting the hypothesis that ComS peptides are directly secreted into the extracellular medium upon synthesis.

(3) $$\begin{array}{l} \frac{dComS^{ext}\left(t\right)}{dt}=\underset{\text{exportation}}{\underset{︸}{r_{out}ComS\left(t\right)}}-\underset{\text{importation}}{\underset{︸}{r_{in}ComS^{ext}\left(t\right)}}-\underset{\text{degradation}}{\underset{︸}{d_{S^{ext}}ComS^{ext}\left(t\right)}}\\ -\underset{\text{growth}}{\underset{︸}{\mu \left(t\right)ComS^{ext}\left(t\right)}} \end{array}$$

Equation (3) describes the time-course evolution of number of ComS^ext^, the secreted mature form of ComS, referred to as XIP (Mashburn-Warren et al., 2010). As noted above, ComS^ext^ peptides were assumed to remain closely associated to the cell surface, as evidenced in the literature (Gardan et al., 2013). This hypothesis is compatible with the timing-device mechanism of competence regulation in *S. thermophilus* proposed by Gardan et al. (2013). Expressing ComS^ext^ values in molecules cell^−1^ is thus relevant in this species. Nevertheless, it is important to note that the specific units (molecules cell^−1^) can be directly converted in volumetric units (molecules ml^−1^) at any time *t* based on the time-dependent value of *X*(*t*) (expressed in number of cells ml^−1^) (see Equation 1). In Equation 3, ComS^ext^ concentration at the cell surface is positively affected by the rate of ComS exportation (same term as above) and negatively affected by the rates of (i) mature ComS^ext^ re-importation in the cytoplasm, (ii) ComS^ext^ degradation in the extracellular medium and (iii) cell division (related to Equation 1). Re-importation of ComS^ext^ through the Opp system was also assumed to depend on a first-order importation constant *r*_*in*_. This simplification can be justified by the fact that in CDM conditions, the Opp transporter is not saturated by nutritive oligopeptides, in contrast to complex medium like M17 (Gardan et al., 2013; Son et al., 2012).

(4) $$\begin{array}{l} \frac{dComS^{*}\left(t\right)}{dt}=\underset{\text{importation}}{\underset{︸}{r_{in}ComS^{ext}\left(t\right)}}-\underset{\text{ComRS formation}}{\underset{︸}{c_{RS}{\left(ComR\left(t\right)ComS^{*}\left(t\right)\right)}^{n}}}\\ -\;\underset{\text{degradation}}{\underset{︸}{d_{S^{*}}ComS^{*}\left(t\right)}}-\;\underset{\text{growth}}{\underset{︸}{\mu \left(t\right)ComS^{*}\left(t\right)}} \end{array}$$

Equation (4) describes the evolution of number of mature intracellular ComS^*^ peptides. In this equation, the importation flux increases linearly with specific ComS^ext^ concentration i.e., the number of ComS^ext^ peptides that are physically associated to one cell. The mechanistic effect of cell density on the importation flux is not modeled in Equation (4) as the cell-cell signaling function of ComS is not considered in the ComRS model. We fit the degradation constants in order to keep the concentrations of ComR and ComS inside the cells close to a 1:1 ratio. The negative term *c*_*RS*_(*ComR*(*t*)ComS^*^(*t*))^*n*^ corresponds to the formation of the ComRS complex, according to the mass action law, as modeled in Son et al. (2012).

(5) $$\begin{array}{l} \frac{dComR(t)}{dt}=b_{R}(t)+\underset{\text{distal regulators}}{\underset{︸}{N(m,\sigma ,t)}}-\underset{\text{ComRS formation}}{\underset{︸}{c_{RS}{\left(ComR(t)ComS^{*}(t)\right)}^{n}}}\\ \;-\;\underset{\text{degradation}}{\underset{︸}{d_{R}ComR(t)}}-\underset{\text{growth}}{\underset{︸}{\mu (t)ComR(t)}} \end{array}$$

In Equation (5), ComR is produced at a time-dependent basal rate *b*_*R*_(*t*) and its expression is activated by unknown distal regulators whose actions are summed up in deterministic Gaussian function *N*(*m*, σ ,*t*): the effects of all the activators/inhibitors of ComR are embedded in this bell-shaped function:

$$\begin{array}{l} N\left(m,\sigma ,t\right)=A\frac{1}{\sigma \sqrt{2\pi }}e^{-\frac{1}{2}{\left(\frac{t-m}{\sigma }\right)}^{2}} \end{array}$$

Formation of the ComRS complex, as well as ComR degradation and cell division, negatively influences *ComR*(*t*) production.

(6) $$\begin{array}{l} \frac{dComRS\left(t\right)}{dt}=\underset{\text{ComRS}\;\text{formation}}{\underset{︸}{\frac{c_{RS}}{2}{\left(ComR\left(t\right)ComS^{*}\left(t\right)\right)}^{n}}}\\ -\;\underset{\text{shut-off by ComZ}}{\underset{︸}{V_{max}^{RSZ}\left(\frac{ComRS\left(t\right)ComZ\left(t\right)}{K_{m}^{RSZ}+ComRS\left(t\right)ComZ\left(t\right)}\right)}}\\ -\;\underset{\text{degradation}}{\underset{︸}{d_{RS}ComRS\left(t\right)}}-\underset{\text{growth}}{\underset{︸}{\mu \left(t\right)ComR\left(t\right)}} \end{array}$$

Equation (6) describes the evolution of active ComRS complexes. A ½ stoichiometric coefficient was introduced to describe the formation rate of the active complex since 2 ComS and 2 ComR molecules are needed to produce 1 ComRS molecule (Fontaine et al., 2013). Production of ComRS is negatively influenced by its sequestration in an inactive complex, ComRSZ (for the role of ComZ, see Equation 8 below), which forms according to a Michaelis-Menten function. As mentioned above, this negative term models the negative feedback loop that operates during competence shut-off (see description of Equation 8 for detailed information).

(7) $$\begin{array}{l} \frac{dComX\left(t\right)}{dt}=b_{X}\left(t\right)+\underset{\text{activation by ComRS}}{\underset{︸}{V_{max}^{X}\left(\frac{ComRS\left(t\right)}{K_{m}^{X}+ComRS\left(t\right)}\right)}}-\underset{\text{degradation}}{\underset{︸}{d_{X}ComX\left(t\right)}}\\ -\;\underset{\text{growth}}{\underset{︸}{\mu \left(t\right)ComX\left(t\right)}} \end{array}$$

Equation (7) has the same structure as Equation (2): ComX is produced at a basal rate *b*_*X*_(*t*), and its expression is activated by the ComRS complex.

(8) $$\begin{array}{l} \frac{dComZ(t)}{dt}=\underset{\text{activation by ComX}}{\underset{︸}{V_{max}^{Z}\left(\frac{ComX{\left(t\right)}^{n_{z}}}{{\left(K_{m}^{Z}\right)}^{n_{z}}+ComX{\left(t\right)}^{n_{z}}}\right)}}-\underset{\text{degradation}}{\underset{︸}{d_{Z}ComZ(t)}}\\ \;-\;\underset{\text{growth}}{\underset{︸}{\mu (t)ComZ(t)}}-\underset{\text{inactivation of ComRS}}{\underset{︸}{V_{max}^{RSZ}\left(\frac{ComRS(t)ComZ(t)}{K_{m}^{RSZ}+ComRS(t)ComZ(t)}\right)}} \end{array}$$

Equation (8) describes the evolution over time of the ComZ protein, which represents the putative actor(s) of the late phase responsible for competence shut-off. We made the hypothesis of a null basal production rate for ComZ, which is assumed to be produced only in the presence of ComX. The model proposes that ComZ associates with ComRS to inactivate it (Figure 1). This hypothesis is based on the shut-off mechanism described in *S. pneumoniae* in which the late protein DprA was shown to interact with ComE~P to block ComE-driven transcription of *comX* (Mirouze et al., 2013).

These eight ODEs form the system S1. At any time *t*, the state *s*(*t*) of the system is determined by the cell density (*X*(*t*), see Equation 1) and the number of each molecular species (ξ_*i*_(*t*), see Equations 2–8), and is defined by the following vector, evolving in the positive orthant (9):

(9) $$\begin{array}{l} s\left(t\right)=\left\{X\left(t\right),\xi_{1}\left(t\right),\xi_{2}\left(t\right),\ldots \xi_{N}\left(t\right)\right\}\in {ℜ}_{+}^{N+1}\text{with }N=7 \end{array}$$

#### Determination of model parameters

The determination of numerical values for the 22 parameters of system S1 was performed in a stepwise fashion, as described in details below. Briefly, system S1 was first simplified by replacing the production terms for *ComR*(*t*), *ComS*(*t*), and *ComX*(*t*) with lumped production terms derived from experimental measurements. This reduced system (termed S2) only contained 15 of the 22 parameters of system S1. In a second step, the 15 parameters of system S2 were determined, according to several mechanistic hypotheses and constraints. In a final step, the lumped, experiment-derived production terms for *ComR*(*t*), *ComS*(*t*), and *ComX*(*t*) were replaced with their mechanistic equivalents in system S1, and the remaining seven parameters were fitted.

##### Step 1—model simplification and inclusion of experimental data on ComS, ComR, and ComX production rates

To determine the time-dependent production rates (due to basal production rates and activation rates) *prod(t)* of variables *ComR*(*t*), *ComS*(*t*), and *ComX*(*t*), we used data on the specific luciferase activities driven from CMDL cultures of LMD-9 derivative strains containing P_*comR*_*-luxAB*, P_*comS*_*-luxAB*, or P_*comX*_*-luxAB* reporter fusions (Fontaine et al., 2013; Supplementary Figure S1B), based on the assumption that the specific production rate of luciferase in a given reporter strain (*prod*_*LuxAB*_(*t*); molecules cell^−1^ min^−1^) is identical to the specific production rate of the peptide or protein under control of the same promoter (*prod*(*t*); molecules cell^−1^ min^−1^):

(10) $$\begin{array}{l} prod\left(t\right)=prod_{LuxAB}\left(t\right) \end{array}$$

In order to determine *prod*_*LuxAB*_(*t*) from the experimentally observed light emission profiles (*L*(*t*); RLU OD${}_{600}^{-1}$) of the reporter strains, the following assumptions were made:

- the observed light emission *L* was assumed to be directly proportional to the amount of luciferase LuxAB:
  (11) $$\begin{array}{l} L\left(t\right)=kLuxAB\left(t\right) \end{array}$$
- the net production rate of luciferase *dLuxAB*(*t*)*/dt* was assumed to be the difference between its absolute production rate (*prod*_*LuxAB*_(*t*)), its degradation according to a constant degradation rate *d*_*LuxAB*_ and its dilution due to cell growth:
  (12) $$\frac{d_{LuxAB}(t)}{dt}=prod_{LuxAB}(t)-(d_{LuxAB}(t)+\mu (t))LuxAB(t)$$

Hence, by combining Equations (10–12), the production rates of ComS, ComR, and ComX can be reformulated as a function of the observed light emission as follows:

(13) $$\begin{array}{l} prod\left(t\right)=\frac{1}{k}\left(\frac{dL\left(t\right)}{dt}+d_{LuxAB}L\left(t\right)+\mu \left(t\right)L\left(t\right)\right) \end{array}$$

For the dilution term, μ(*t*) was calculated from the experimental growth curve of *S. thermophilus* LMD-9 in CDML, as described in Equation (1) (Supplementary Figure S1A). The degradation rate of luciferase *d*_*LuxAB*_ was set to 0.015 min^−1^, as observed experimentally in *S. thermophilus* (data not shown). The value of the proportionality constant *k* (2.197 10^−4^ RLU molecule^−1^) was chosen so as to mimic the experimentally observed light emission profiles of the three reporter strains (LMD-9 derivatives containing P_*comS*_-*luxAB*, P_*comX*_-*luxAB*, and P_*comR*_-*luxAB*). Parameter *k* was thus assumed to be an intrinsic property of the luciferase LuxAB, independent on the promoter driving its expression. For determining *k*, an additional constraint was set on the system, by imposing a maximal production rate for ComS (*prod*_*S*_*(t)*) of 20 molecules per minute, in accordance with the estimated maximal synthesis rate of small peptides (Bolouri and Davidson, 2003).

##### Step 2—determination of parameters for system S2

Since the seven parameters of the modeled production rates for *ComS(t), ComS(t)*, and *ComX(t)* are hidden behind the experimental production rates, the number of parameters to estimate in system S2 is only *m* = 15, instead of 22 in system S1. Based on mechanistic assumptions and literature information, specific values were assigned to 2 of the 15 parameters:

- The Hill coefficient for formation of the ComRS complex was set to *n* = 2.5, according to Son et al. (2012), to capture the dimeric character of the complex (Fontaine et al., 2013).
- The degradation rate of ComX (*d*_*X*_) was set to 0.2 min^−1^, according to Karlsson et al. (2007). This corresponds to a half-life of around 3 min, which is in the same range as the experimental half-life estimated for ComX in *S. pneumoniae* (~4 min) (Luo and Morrison, 2003).

In addition, the degradation rates of the other variables were related by the following inequality:

$$\begin{array}{l} 0.01{\text{min}}^{-1}<d_{R}=d_{RS}=d_{Z}<d_{S^{*}}=d_{Sext}<0.69{\text{min}}^{-1} \end{array}$$

which is the mathematical expression of imposing (i) that the half-lives of the peptides/proteins should lie between around 1 min and 1 h, and (ii) that, due to the respective molecular weights of the molecules, the degradation of small peptides (ComS^ext^ and ComS^*^) is faster than the degradation of bigger proteins. Because of their similar size (~10 amino acids; Gardan et al., 2013), the degradation rates of ComS^ext^ and ComS^*^ were assumed to be identical. The possible influence of ComS^*^ on the stability of the ComRS complex was neglected, as previously assumed by Voigt et al. (2005) for a similar protein-peptide complex. Hence, the degradation rates of ComR and ComRS were assumed to be identical. Finally, since ComZ is a hypothetical protein, its degradation rate cannot be anticipated on rational grounds: by default, it was constrained to be identical to that of ComR and ComRS.

**Figure 2:**
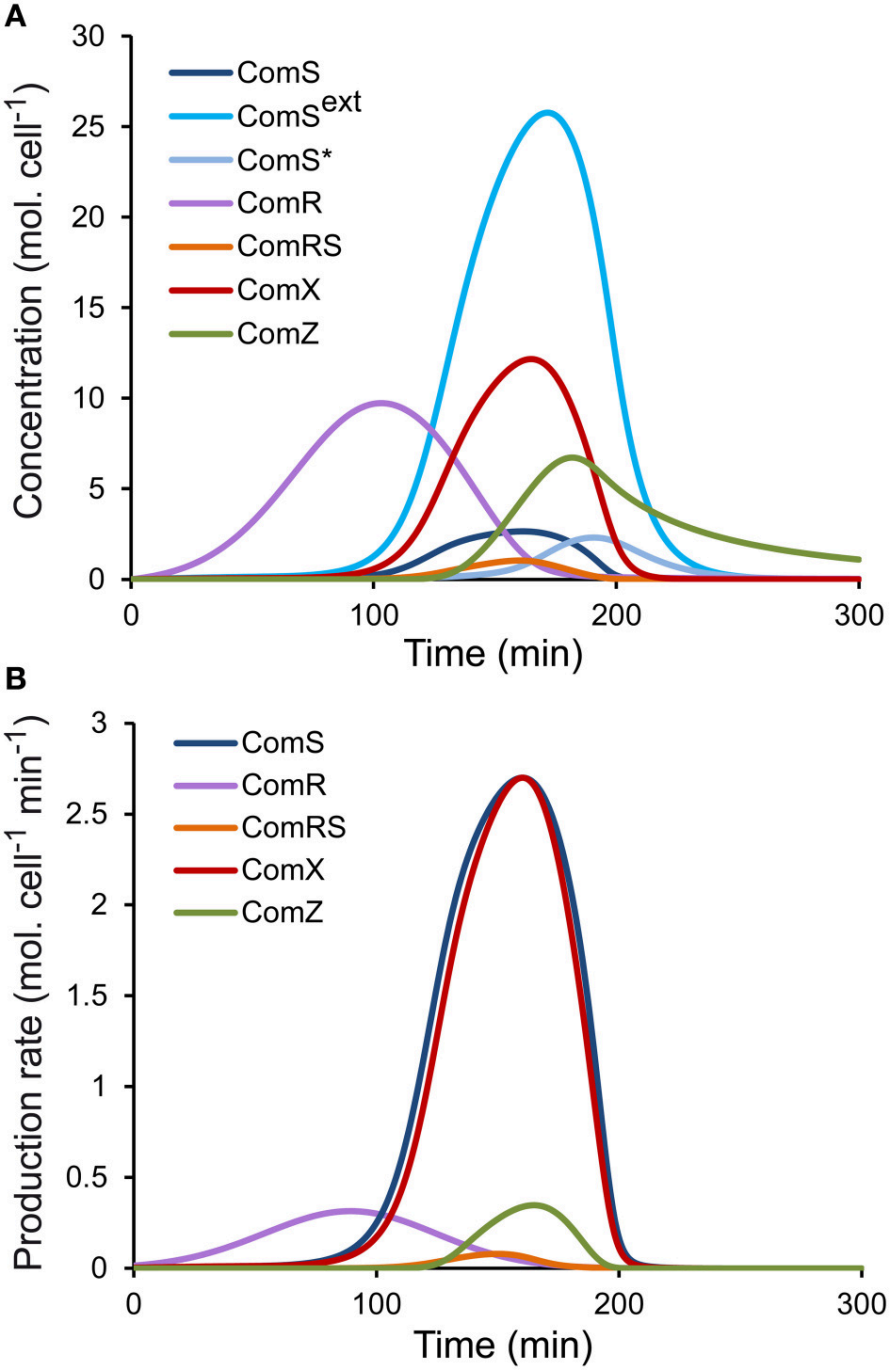
**Simulated profiles of molecular species of the ComRS model**. Time-course evolution of **(A)** the concentrations (molecules (mol.) cell^−1^) and **(B)** the production rates [molecules (mol.) cell^−1^ min^−1^] of the modeled molecular species in a WT LMD-9 strain in CDML growth conditions.

Numerical values for the remaining ten parameters of system S2 were set with the requirement that the resulting model adequately describes the following experimentally observed behaviors:

- Since we initially assumed that luciferase production in the reporter strains is proportional to the production of the corresponding peptides (see Step 1—Model Simplification and Inclusion of Experimental Data on ComS, ComR, and ComX production Rates), two qualitative requirements were set on the simulated profiles of intracellular concentrations of ComR, ComS, and ComX. First, the time sequence of the initial increase in concentration of the three peptides/proteins (Figure 2A) must qualitatively match the time sequence of the onset of light production (Supplementary Figure S1B), i.e., if luciferase activity is first detected in the P_*comR*_*-luxAB* reporter strain, then the simulated ComR accumulation must start before that of ComS and ComX. The production of the late competence protein ComZ must be delayed compared to ComX, as observed experimentally between the activation of P_*comX*_-*luxAB* and P_*comGA*_-*luxAB*, another late *com* gene (data not shown). Second, the time sequence of the maximum intracellular concentration of the three peptides/proteins (Figure 2A) (null derivative, where the production term equals the consumption, degradation, and dilution terms) must qualitatively match the time sequence of experimentally observed maximum luciferase activity in the corresponding reporter strains (Supplementary Figure S1B). When we compare Figure 2A (modeled kinetics) and Supplementary Figure S1B (experimental kinetics), we see that the two qualitative requirements are globally satisfied. The shape and amplitudes of the simulated curves must however not necessarily match those of luciferase activity, since except for the dilution term, consumption terms for ComR, ComS, and ComX differ between each other and from the degradation term of LuxAB.
- It was required that in the modeled system, the concentration of ComX in simulated wild-type LMD-9 cells is not at saturation. In other words, we imposed that the maximum concentration of ComX in simulated cells can be further increased upon addition of extracellular ComS (i.e., non-zero initial values of the variable *ComS*^*ext*^(*t*)), in agreement with experimental observations showing that natural transformation frequencies and the activity of P_*comX*_*-luxAB* in wild-type LMD-9 can be further increased by addition of the synthetic mature ComS peptide ComS_17-24_ (Fontaine et al., 2010a and data not shown).

##### Step 3—determination of the remaining parameters for system S1

Using the 15 parameter values determined in the previous step, we then moved back to system S1, and replaced the experimentally-derived production rates *prod*_*R*_(*t*), *prod*_*S*_(*t*), and *prod*_*X*_(*t*) with their explicit mechanistic equivalents in Equations (5), (2), and (7) respectively. In all three cases, the mechanistic production terms are composed of a basal production rate term and an activation term.

The time-dependent basal expression rate of ComS (*b*_*S*_(*t*)) was derived from an experimental dataset of luciferase activity in a CDML culture of an LMD-9 Δ*comR* strain containing a P_*comS*_*-luxAB* reporter fusion (Supplementary Figure S1C). Indeed, since the deletion of *comR* prevents formation of the ComRS complex, it therefore suppresses the activation of ComS production by ComRS, and the remaining luciferase activity thus represents the basal activity of promoter P_*comS*_ (Fontaine et al., 2013). Specific luciferase activity (RLU OD${}_{600}^{-1}$) was converted into the basal production rate *b*_*S*_(*t*) (molecules cell^−1^ min^−1^) as described in step 1 for determination of the production rates of ComR, ComS, and ComX, using the formula:

(14) $$\begin{array}{l} b_{S}\left(t\right)=\frac{1}{k}\left[\frac{dL_{\Delta comR}\left(t\right)}{dt}+\left(d_{Lux}+\mu \left(t\right)\right)L_{\Delta comR}\left(t\right)\right] \end{array}$$

where *L*_Δ*comR*_ is the luciferase activity of the Δ*comR* strain containing a P_*comS*_*-luxAB* reporter fusion. We used the same value of 2.197 × 10^−4^ for the proportionality constant *k* for ComR, ComS, and ComX. For simplicity, we also assumed that the basal expression rates of ComR and ComX are identical to *b*_*S*_*(t)*: *b*_*R*_*(t)* = *b*_*X*_*(t) = b*_*S*_(*t*). It is worth noting that these basal production functions are needed in the model to initiate the competence processus from zero initial concentrations. But their amplitudes remain negligible, with respect to the activation terms, all along the culture. Therefore, the convenient approximation of taking identical functions is not a really restrictive condition here.

Numerical values were then set for the seven additional parameters of system S1, which define the activation rates of ComS (*K*${}_{m}^{S}$ and *V*${}_{max}^{S}$) and ComX (*K*${}_{m}^{X}$ and *V*${}_{max}^{X}$) by ComRS and the activation rate of ComR by distal regulators (*m*, σ, *A*). Besides the qualitative requirements already used for system S2 (see Step 2—determination of Parameters for System S2), we also imposed the requirement that the simulated profiles of ComR, ComS, and ComX production rates (basal rate + activation rate) (Figure 2B) are similar in shape to the corresponding luciferase production rates (*prod*_*L*_(*t*)) deduced from experimental observations (Supplementary Figure S2). Constraints were also imposed on the parameter values for *K*${}_{m}^{S}$, *K*${}_{m}^{X}$, *V*${}_{max}^{S}$, and *V*${}_{max}^{X}$. Since our model does not explicitly deal with processes such as transcription and translation, *K*_*m*_ and *V*_*max*_ values cannot be directly compared to existing biological values of other systems. Nevertheless, we constrained those parameters to biologically relevant values, i.e., in the nM range as reported by Jabbari et al. (2011). We also imposed that *K*${}_{m}^{S}$ is lower than *K*${}_{m}^{X}$ because the binding affinity of ComR to its DNA motif (ComR-box) was previously predicted to be higher in P_*comS*_ compared to P_*comX*_ (Fontaine et al., 2013).

The resulting parameter values for the 22 parameters of system S1 are provided in Table 2.

#### Simulation protocols

All computations and simulations were done using the MATLAB (R2012a) software. In particular, the ode23 routine was used for the integrations over time, with a null initial condition (except for dose-response simulations). Our full MATLAB source code is available as Supplementary Data Sheet 1. Experimental data necessary to run the code are available as Supplementary Data Sheet 2.

The mean behavior of a wild-type cell was simulated *in silico* by integrating system S1 over time (from *t* = 0 to *t* = 400 min), with the parameters listed in Table 2. A null initial condition was used: at *t* = 0, all cells were assumed to be devoid of any molecule of the modeled variables: *s*(*t*_0_) = {*X* (*t*_0_), 0, 0, 0, 0, 0, 0,0}.

##### Simulation of knocked-out strains

To mimic a strain deleted for a gene of interest, the corresponding equation was set to zero in S1, and that modified S1 system was integrated over time with the same ode23 routine.

##### Simulation of dose-responses to ComS

To mimic the supplementation of ComS_17-24_ peptides in the growth medium, the variable *ComS*^*ext*^(*t*) was given a non-zero initial value, between 0 and 2000 molecules per cell, at *t* = 0: *s*(*t*_0_) = {*X* (*t*_0_), 0, *V*_0_, 0, 0, 0, 0,0}.

To study the consequence of decreasing the affinity between ComR and ComS on dose-response, the complex formation constant *c*_*RS*_ was decreased by a factor ranging from 10^1^ to 10^5^. To simulate supplementation of ComS_(17-24)_ at different times T of growth, the initial condition of *ComS*^*ext*^(*t*) was set to 0 until time T, then at time T (*T* = [30, 60, 90, 120, 150, 180, 210] in min), an initial condition of ComS^ext^ molecules (i.e., 150 molecules per cell) was set. For each condition (various ComS^ext^ concentrations or induction times), the system S1 was integrated until *t*_*end*_ = 400 min with the ode23 routine and the maximal amount of ComX molecules per cell reached over time was computed.

##### Simulation of ComR or ComS overproduction

The different levels of ComR or ComS production were simulated by replacing their respective basal rate *b*_*R*_(*t*) and *b*_*S*_(*t*) by a constant value R: R = max(*b*(*t*)) IF.

When not otherwise stated, the induction factor (IF) was set to 50.

##### Sensitivity analysis

Our sensitivity analysis was performed by varying the value of each parameter within an interval ranging of 0.1–10 times its assigned value (listed in Table 2) unless otherwise stated. The maximal value of ComX obtained over time in this range was then computed.

## Results

### Modeling the control of the master competence regulator ComX by ComRS

In CDM conditions, the ComRS signaling system dominantly controls *comX* expression in *S. thermophilus* (Fontaine et al., 2013) and *S. mutans* (Mashburn-Warren et al., 2010; Son et al., 2012; Reck et al., 2015). We thus hypothesized that the modeling of the ComRS regulatory pathway would recapitulate regulation of ComX production in those growth conditions.

The LMD-9 strain of *S. thermophilus* was chosen as model strain since the competence regulating network of this strain has been extensively studied and a range of experimental data are available on the expression of key competence genes in wild type (WT) and competence-deficient backgrounds (Fontaine et al., 2010a, 2013; Gardan et al., 2013). Importantly, LMD-9 is also naturally competent in CDM, in contrast to most strains tested so far, where exogenous addition of micromolar concentrations of mature ComS peptides is necessary to reach high levels of competence (Fontaine et al., 2010b).

In CDM growth conditions, competence for natural transformation in *S. thermophilus* is a transient phenomenon. To take into account this expression dynamics, ordinary differential equations (ODEs) were used to model the time-course evolution of (known) principal molecular species involved in *comX* induction and shut-off (Figure 1). The ODE formalism is widely used in systems biology and presents several advantages: it allows to set up structural and dynamical models with an adjustable level of detail, allowing to capture various physical phenomena in a transparent way, to use experimental time-course data, and to test different hypotheses easily (de Jong, 2002).

The modeled peptide/proteins (expressed in number of molecules per cell) are: (i) cytoplasmic prepeptides ComS; (ii) mature secreted pheromones ComS^ext^ (also named XIP); (iii) reimported mature pheromones ComS^*^; (iv) unbound cytoplasmic ComR transcriptional regulators; (v) active ComRS complexes; (vi) competence sigma factor ComX and (vii) ComZ, which embeds the actor(s) controlled by ComX that are responsible for competence shut-off. ComZ is assumed to inhibit the activity of ComRS by forming a ternary ComRSZ complex (Figure 1). An equation modeling the population cell density over time (*X*(*t*), expressed in number of cells per ml) was also added to the system, to couple competence development to the growth process. At any time *t*, the state of the system consequently depends on the cell density.

The numerical values of the *n* (*n* = 22) parameters of the eight ODE equations were empirically determined on the basis of (i) literature; (ii) mechanistic hypotheses; and (iii) experimental data (see Materials and Methods for detailed information). The latter consist of the growth parameters of strain LMD-9 (Supplementary Figure S1A) and specific luciferase activities driven from promoters P_*comX*_, P_*comS*_, and P_*comR*_ in WT and ComR^−^ reporter strains grown in CDM conditions (Supplementary Figures S1B,C).

#### Meta-hypotheses

Three meta-hypotheses were assumed to build the mathematical model of the ComRS regulatory cascade. First, our model describes the behavior of a homogenous population of cells in which all the individuals are assumed to act exactly the same way, i.e., we made a mean-field approximation of individual cells. The modeled behavior can thus be seen as the mean behavior over a large sample of cells, neglecting the intercellular variations. This simplification was made because the experimental data used to calibrate the model parameters derive from luciferase activities measured at the population scale level (see Materials and Methods). This explains why the values of some parameters are not whole numbers in our simulations (Table 2). Second, we used the quasi-steady-state assumption for mRNA dynamics, hence focusing on the protein level only. This simplification is generally accepted because RNAs fluctuate much more rapidly, and hence, can be assumed to be in quasi-steady-state compared to the slower varying protein level. Third, we assumed that the transcription and translation steps are instantaneous: as previously discussed (Young and Bremer, 1975; Proshkin et al., 2010), delays can be neglected in prokaryotes due to the lack of compartmentalization.

### Model validation

To get confident in our parameter setting and in the predictive power of our model, the output of different sets of simulation were compared to existing or original experimental data. The chosen simulations aimed to test reactivity, dynamics and topology of the system.

#### Reactivity to ComS induction

**Figure 3:**
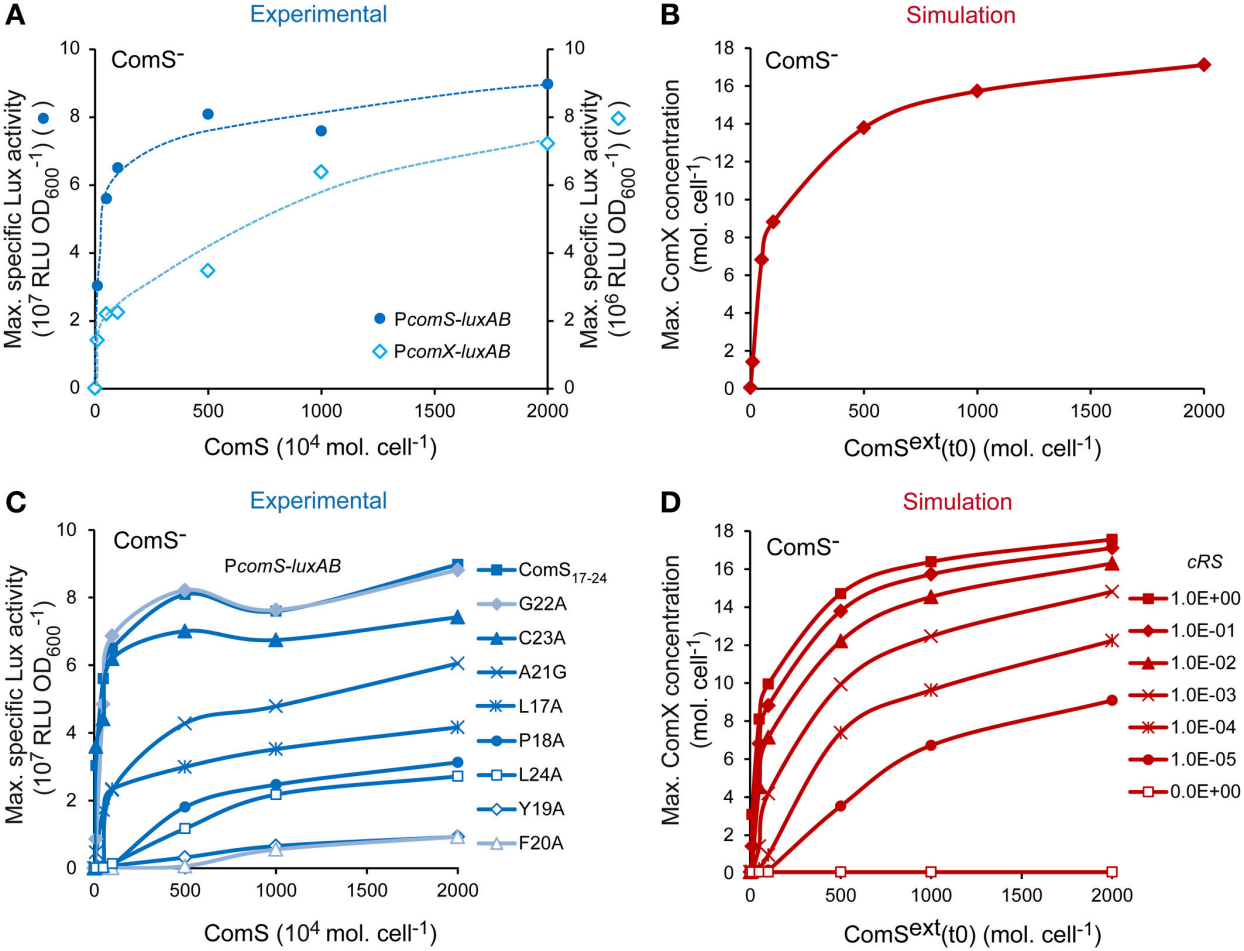
**Validation of the reactivity of the ComRS model. (A)** Maximum specific luciferase activity (RLU OD${}_{600}^{-1}$) driven by P_*comX*_ and P_*comS*_ in the ComS^−^ strains LF118 and LF134, respectively, in response to increasing ComS_17-24_ concentrations. **(B)** *In silico* computation of the maximum ComX concentrations (molecules (mol.) cell^−1^) reached in response to increasing initial ComS^ext^ concentrations (mol. cell^−1^) in a simulated ComS^−^ background. **(C)** Impact of the systematic replacement of each amino acid residue of ComS_17-24_ with Alanine on the reactivity of the P_*comS*_-*luxAB* fusion in ComS^−^ strain (LF134). Graph shows the maximum RLU OD${}_{600}^{-1}$ measured after addition of increasing peptides concentrations **(D)** *In silico* impact of decreasing the affinity between ComR and ComS on the maximum ComX concentrations (mol. cell^−1^) reached. This was done by decreasing the value of the ComRS complex formation rate constant *c*_*RS*_ by 10-fold steps. Concentrations of ComS^ext^ (mol. cell^−1^) and ComS_17-24_ (nM) used are 0, 10, 50, 100, 500, 1000, and 2000. To enable comparison between *in silico* and *in vitro* experiments, experimental nanomolar units of ComS_(17-24)_ were converted in 10^4^ mol. cell^−1^ by assuming a density of 5 × 10^7^cells ml^−1^ at the moment of peptide addition (i.e., after 1 h of incubation in CDML). **(A,C)** The results of one representative experiment are shown.

It was previously shown that the competence default of ComS^−^ derivatives or poorly transformable strains may be supplemented by exogenous synthetic ComS_17-24_ peptides (Fontaine et al., 2010a,b, 2013). This behavior is typically observed in pheromone-based signaling systems (Sturme et al., 2002). One first set of validation consisted thus in testing if our model responded similarly to extracellular mature ComS (experimental synthetic ComS_17-24_ or simulated ComS^ext^) in a ComS^−^ background. To this end, the ComS dose-response curves of experimental and simulated ComS^−^ backgrounds were compared. Experimental curves were obtained by measuring the maximum luciferase activity emitted by the ComS^−^ P_*comX*_-*luxAB* and P_*comS*_-*luxAB* reporter strains in response to increasing synthetic ComS_17-24_ concentrations. The simulated curves were obtained by computing the maximum number of ComX molecules per cell obtained by integrating our system with an increasing initial concentration for ComS^ext^. To mimic a ComS^−^ background in our model, Equation (2), describing the production of ComS, was set to zero (see Materials and Methods). Experimental molar ComS_17-24_ concentrations were converted into molecules per cell to facilitate the comparison. As respectively illustrated in Figures 3A,B, experimental (blue) and simulated (red) curves are both qualitatively and quantitatively comparable. Indeed, the reactivity of the *in silico* and *in vivo* systems is in the same range: ComX concentrations were respectively induced ~280- and 560-fold by saturating peptide concentrations. Interestingly, the minimal pheromone concentration that is needed to experimentally turn on the system is ~10^4^-fold higher than the concentration needed to activate the system *in silico*. This suggests that all the synthetic ComS_17-24_ peptides that are added to the cell culture are not effectively reimported inside the cells. This could be due to ComS degradation by cell surface proteases (e.g., HtrA), as described for other pheromones (Cassone et al., 2012), ComS aggregation in the growth medium or a limitation in the diffusion of ComS molecules through the cell wall.

Previous studies showed that the reactivity of streptococci to ComS induction is species-specific, suggesting that it is strongly dependent on the specificity of the ComR-ComS interaction. In S. *thermophilus*, some mutations in ComS_17-24_ were shown to decrease its biological activity, probably by affecting the formation of active ComRS complexes (Fontaine et al., 2013). Indeed, the efficiency of ComS_17-24_ variants to induce P_*comS*_-*luxAB in vivo* (see the dose-response curves on Figure 3C) is strongly related to its ability to induce the formation of ternary P_*comS*_-ComRS complexes *in vitro* (Supplementary Figure S3). A second set of simulations aimed thus to test whether ComX production was similarly dependent on the rate of ComRS complex formation in our model. The value of the ComRS complex formation rate constant *c*_*RS*_ was decreased (see Materials and Methods) and dose-response curves were simulated by computing the maximum number of ComX molecules produced in a ComS^−^ simulated cell (Figure 3D). As expected, our model was able to simulate the effect of ComS mutations that affect the reactivity of ComRS *in vivo*: the amplitudes and profiles of simulated dose-response curves resemble those observed with mutated ComS_17-24_ variants in the reporter strain (compare Figure 3C and Figure 3D).

#### Dynamics of induction

**Figure 4:**
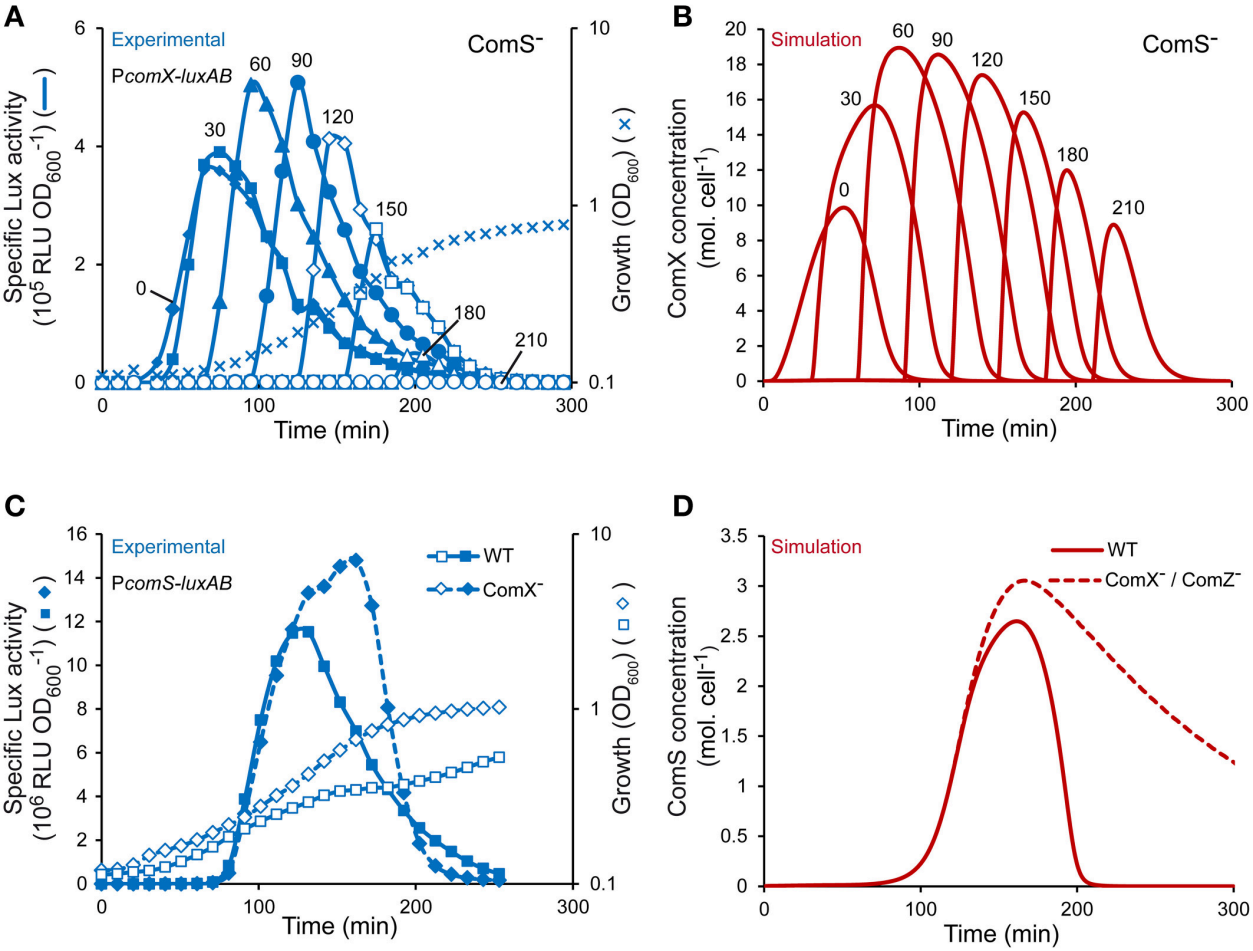
**Validation of the dynamics and topology of the ComRS model. (A)** Specific activity of P_*comX*_ upon ComS_17-24_ addition at different times of growth of a ComS^−^ luminescent reporter strain (TIL1391). The curves show the specific luciferase activity (RLU OD${}_{600}^{-1}$) of the reporter strain measured directly after addition of 1 μM ComS_17-24_ at different times of growth. The growth curve (OD_600_) of the reporter strain grown in CDML in absence of ComS_17-24_ is shown. Data were adapted from Gardan et al. (2013). **(B)** Modeled kinetics of ComX concentrations (molecules (mol.) cell^−1^) in a ComS^−^ background obtained by simulating the addition of ComS^ext^ (150 mol. cell^−1^) at different times of growth. The time T expressed in min (*T* = 0, 30, 60, 90, 120, 150, 180, and 210) at which ComS_17-24_ and ComS^ext^ were experimentally and *in silico* added, respectively, is indicated above each curve. **(C)** Comparison of the kinetics of the specific activity of P_*comS*_ between WT (LF121) and ComX^−^ (LF122) reporter strains (RLU OD${}_{600}^{-1}$) in CDML growth conditions. **(D)** Comparison of modeled kinetics of ComS concentrations (mol. cell^−1^) between WT and ComX^−^ or ComZ^−^ strains. **(A,C)** The results of one representative experiment are shown.

In CDM medium, the timing of induction by mature ComS peptides (i.e., ComS_(17-24)_) was previously shown to be critical to reach optimal competence levels (Gardan et al., 2013; Figure 4A). To test the dynamics of ComX production over time in our model, we simulated the supplementation of mature ComS peptides (i.e., ComS^ext^) at different moments during growth of a ComS^−^ strain (see Materials and Methods). Maximum ComX concentrations computed at each induction time is in good accordance with experimental data (compare Figure 4A and Figure 4B) (Gardan et al., 2013). Indeed, ComX production is inducible at all times of growth but both the rate and amplitude are weaker at time 0 and reach an optimum between 60 and 90 min after inoculation. The apparent discrepancy between modeled and experimental output when cells reach the stationary phase (i.e., at induction times of 180 and 210 min) is probably due to the loss of luciferase activities resulting from a depletion of FMNH_2_ (data not shown and Gardan et al., 2013).

#### Topology of the ComRS model

The topology of model S1 was tested by simulating the behavior observed in a ComX^−^ background, in which the activation of ComRS-controlled early gene *comS* is stronger and lasts longer, as a probable consequence of shut-off deregulation (Figure 4C) (Boutry et al., 2013). The ComX^−^ background was simulated by setting equation describing the evolution of *ComX*(*t*) to zero in the model (Equation 7). As expected, the kinetics and amplitude of the simulated evolution of ComS concentrations in a ComX^−^ background are similar to those of the luciferase activity reporting the activation of the P_*comS*_ promoter in the ComX^−^ reporter strain LF122 (compare Figure 4C and Figure 4D). Competence shut-off is indeed delayed in both cases. It is noteworthy that this delay appears shorter in experiments compared to simulations. However, this difference is artefactual since the well-documented loss of luciferase activity in the stationary phase (Duncan et al., 1994; Koga et al., 2005) is largely responsible for the decrease in *comS* expression (Figure 4C). The delayed drop in simulated ComS concentration (Figure 4D) solely results from the negative terms modeled in the corresponding equation, which account for ComS exportation, degradation and cell division (see Materials and Methods).

### ComR abundance limits the competence level of *S. thermophilus* LMD-9

**Figure 5:**
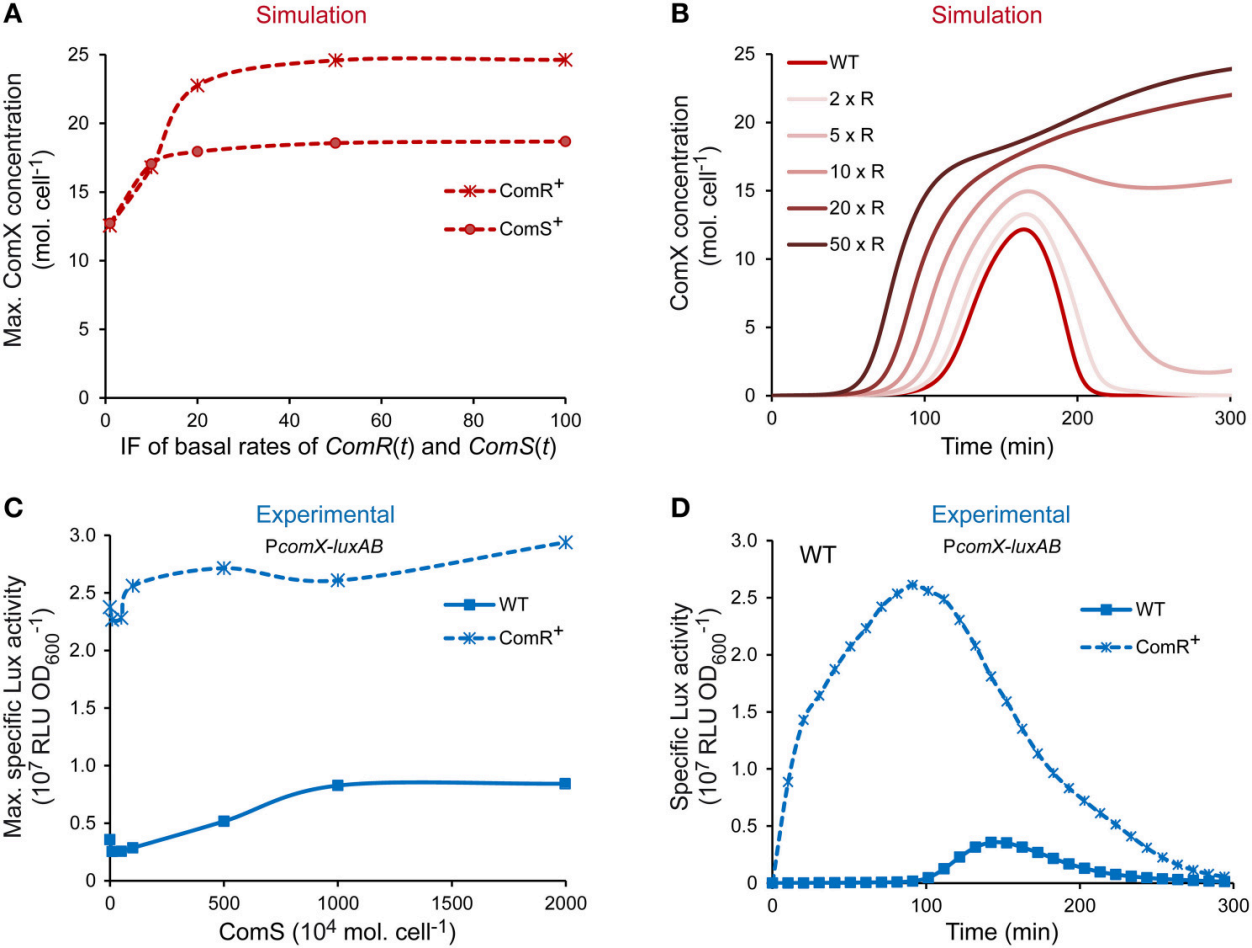
**Effect of ComR and ComS overproduction on the level and timing of competence development in strain LMD-9. (A)** Maximum ComX concentration [molecules (mol.) cell^−1^] computed by increasing the production rates of ComR and ComS. **(B)** Kinetics of ComX concentration (mol. cell^−1^) computed by increasing the production rate of ComR. Production rates were increased by multiplying the maximal basal production rate of variables *ComR*(*t*) and *ComS*(*t*) by a constant inducing factor (IF). IF used in **(A,B)** are [1, 10, 20, 50, 100] and [1, 2, 5, 10, 20, 50] (IF × R), respectively. **(C)** Comparison of the maximum specific luciferase activity of CB001 reporter strains (P_*comX*_-*luxAB*) containing either an empty control vector (CB001 [pMG36eT]; WT) or the constitutive *comR* overexpression vector (CB001 [pMGcomRstrep]; ComR^+^) in response to increasing ComS_17-24_ concentration. ComS_17-24_ concentrations (nM) used are 0, 10, 50, 100, 500, 1000, and 2000. Nanomolar units were converted in 10^4^ mol. cell^−1^ (see legend of Figure 3). **(D)** Comparison of the kinetics of specific P_*comX*_ activity in reporter strains containing either the *comR* overexpression vector (CB001 [pMGcomRstrep]; ComR^+^) or a control vector (CB001 [pMG36eT]; WT) in absence of ComS_17-24_. In all experiments, strains were inoculated in CDML at an OD_600_ of 0.05. Growth (OD_600_) and luciferase activity (RLU) were directly monitored until strains reached the stationary phase. **(C,D)** The results of one representative experiment are shown.

The level of competence of *S. thermophilus* LMD-9 is directly dictated by the amount of ComX molecules produced (Fontaine et al., 2010a; Boutry et al., 2013). The spontaneous activation of *comX* expression in that strain is not at its maximum in CDM conditions since it may be further increased by adding saturating concentration of ComS (Fontaine et al., 2010a,b and data not shown). The ComRS model was used to determine which known actor limits *comX* expression in strain LMD-9 and, consequently, the competence level. To this end, a sensitivity analysis was exploited to identify the variable(s) and/or parameter(s) of the ComRS model having the most important impact on the amplitude of the model output. Parameters were grouped according to their role in equations (Supplementary Figure S4). Most sensitive parameters influence the specific amount of variables ComS (all peptidic forms: precursor, exported, and reimported mature peptide) and ComR. They account for their degradation rate (Supplementary Figure S4G), the rate of ComRS complex formation (Supplementary Figure S4B), and production rates (Supplementary Figures S4C,E,F,H). To further compare how ComS and ComR concentrations impact the competence level of LMD-9 cells, we tested *in silico* the effect of a ComS vs. ComR overproduction (respectively designated ComS^+^ and ComR^+^ in Figure 5A) on the maximum number of ComX molecules. Simulations were performed by multiplying the maximum basal production rate of ComR and ComS by a constant induction factor (IF) ranging from 1 to 100 (see Materials and Methods).

At IF ranging from 1 to 15, the maximum cellular ComX concentration increases similarly in both ComS^+^ and ComR^+^ simulated cells (Figure 5A). However, stronger induction resulted in a ~30% higher output in the case of ComR overproduction. At saturation, a 2- vs. 1.5-fold increase of ComX concentration is observed in ComR^+^ vs. ComS^+^ cells. Importantly, similar ouputs were obtained when the value of each parameter was varied separately within an interval ranging of 0.5–2 times its assigned value, indicating that this prediction is robust (Supplementary Figure S5).Those results suggest that increasing the cellular concentration of ComR vs. ComS will lead to stronger levels of *comX* expression in strain LMD-9. Interestingly, simulations also predict that the abundance of ComR controls the kinetics of competence in CDM. Indeed, the increased production rate of ComR is directly correlated to an earlier induction of ComX production (Figure 5B). To test those predictions experimentally, the competence level and kinetics of a WT and a ComR^+^ strain, which contains a *comR* expression vector (Boutry et al., 2013), were compared. The effect of increasing ComS abundance in both backgrounds was tested by monitoring P_*comX*_ in response to increasing ComS_17-24_ concentrations. Results obtained (Figures 5C,D) show that compared to WT cells, P_*comX*_ activity in ComR^+^ cells is (i) no more inducible by ComS_17-24_, indicating that ComS does not limit competence in those conditions; (ii) is stronger at all ComS_17-24_ concentrations tested (Figure 5C); and (iii) increases from the beginning of growth and is thus no more dependent on the growth phase (Figure 5D).

Altogether, our results strongly suggest that ComR abundance is the major limiting factor which controls the level of *comX* expression and the kinetics of competence induction in the naturally transformable strain LMD-9.

### Low competence of strain LMG18311 is due to weak *comR* expression

**Figure 6:**
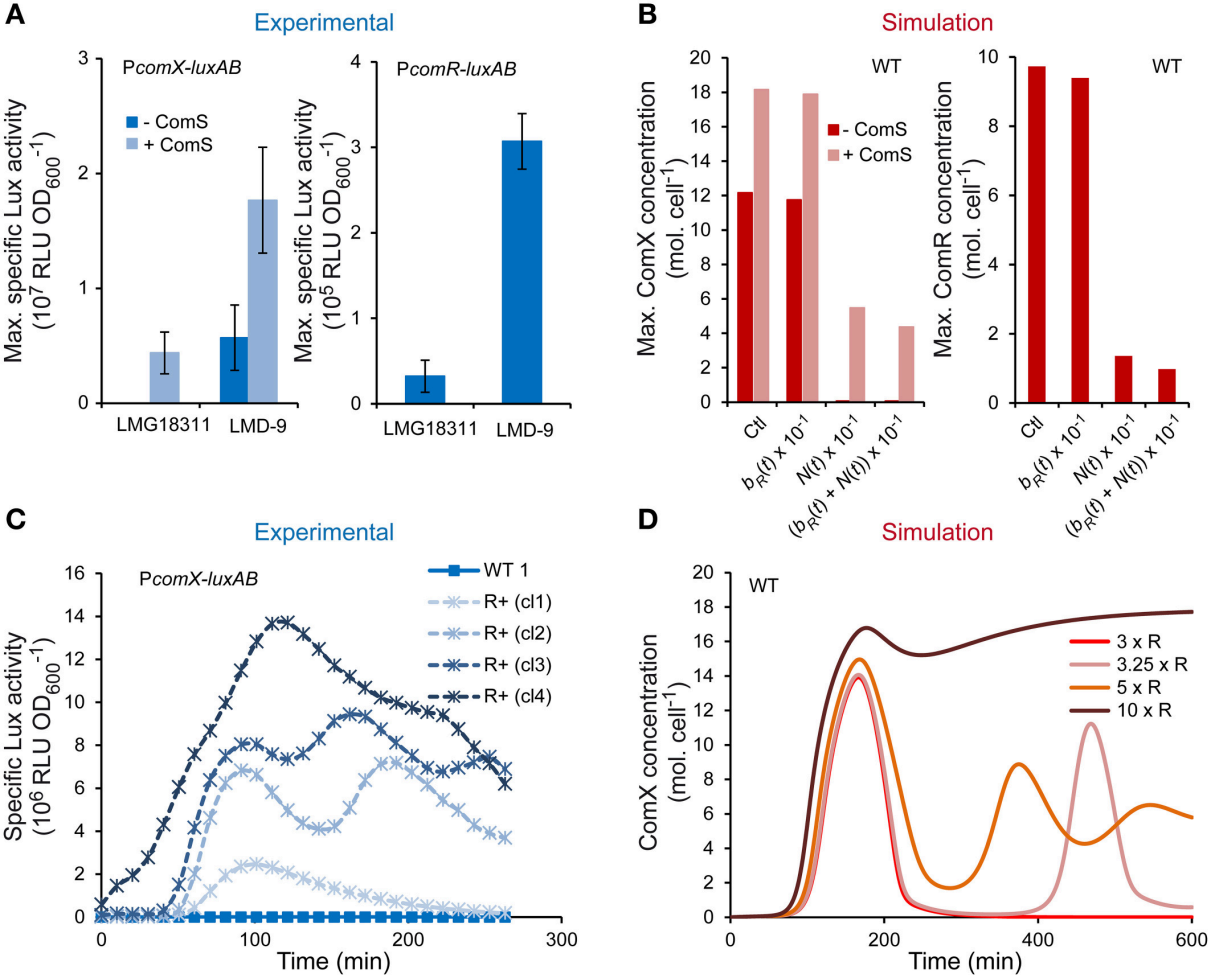
**Role of ComR abundance on the competence level of ***S. thermophilus*** LMG18311. (A)** Maximum specific luciferase activity driven by promoters P_*comX*_ (left panel) and P_*comR*_ (right panel) in respective reporter strains CB009 and LF146 grown in CDML. The symbols (− ComS) and (+ ComS) respectively indicate that no ComS_17-24_ and 1 μM ComS_17-24_ was added 1 h after inoculation. The values are means from triplicate experiments ± standard errors of the means (SEM). **(B)** Maximum concentrations [molecules (mol.) cell^−1^] of ComX (left panel) and ComR (right panel) computed by integrating the system with parameters values of ComR production rate, i.e., *b*_*R*_(*t*) + *N*(*t*) of system S1 (Ctl) (see Table 2), with a 10-fold lower ComR basal production rate (*b*_*R*_(*t*) × 10^−1^), with a 10-fold lower ComR activation rate (*N*(*t*) × 10^−1^) and with a 10-fold lower total ComR production rate ((*b*_*R*_(*t*) + *N*(*t*)) × 10^−1^). ComX molecules were computed by setting an initial condition of ComS^ext^ equal to zero (- ComS) or equal to 150 mol. cell^−1^ (+ ComS). **(C)** Kinetics of specific luciferase activity (RLU OD${}_{600}^{-1}$) by representative clones (cl1, cl2, cl3, and cl4) of the LMG18311 derivative reporter strains CB009 (P_*comX*_-*luxAB*) containing an empty plasmid (CB009 [pMG36eT]; WT) or the constitutive *comR* overexpression vector (CB009 [pMGcomRstrep]; ComR^+^). **(D)** Consequences of increasing the maximum ComR basal production rate by a 3 (3 × R), 3.25 (3.25 × R), 5 (5 × R), and 10-fold (10 × R) factor on modeled kinetics of ComX concentrations (mol. cell^−1^). These induction factors were selected to illustrate the transition between mono-, bi-, and tri-phasic kinetics profiles.

The ability of *S. thermophilus* to spontaneously turn on high competence levels under CDM growth conditions seems restricted to rare strains (Fontaine et al., 2010b). Since all of the key early competence genes to date are encoded in the genome of the poorly transformable strains, we assumed that differences in competence efficiency between *S. thermophilus* strains could be due to different production levels of actors of the ComRS signaling pathway. As ComR abundance was shown to limit competence in strain LMD-9, we studied the activity of P_*comR*_ and P_*comX*_ in the poorly transformable LMG18311 strain. As expected, maximum P_*comX*_ activity is very low in LMG18311 but might be increased 345-fold upon ComS_17-24_ supplementation (Figure 6A). Similar results were obtained for P_*comS*_ activity (data not shown), which confirms that similarly to other weak competent strain, ComS production is limiting in strain LMG18311. Interestingly, P_*comR*_ activity is also ~10-fold lower in strain LMG18311 compared to strain LMD-9 (Figure 6A).

The ComRS model was next used to test whether the observed difference in *comR* expression accounts for low expression of *comX*. To mimic strain LMG18311, the production rate of *ComR*(*t*) was divided by 10. The latter is modeled as the sum of rates due to ComR basal production (*b*_*R*_(*t*)) and activation/repression by -as yet- unidentified regulator(s) (*N*(*t*)). As shown on Figure 6B, simulations done after dividing both terms reproduced the behavior of strain LMG18311: maximum cellular ComX concentration reaches high levels only in presence of initial ComS^ext^ concentrations. In addition, a similar four-fold difference in simulated and experimental outputs (respectively maximum ComX concentration and P_*comX*_ activity) was observed between strains LMG18311 and LMD-9 in ComS-induced conditions. Similar results were obtained when the rate of *ComR*(*t*) activation/repression solely was divided in the ComRS model, while it was not the case when only the basal rate was divided by 10. This suggests that the contribution of ComR basal production to ComX production is minor in *S. thermophilus* (Figure 6B).

In conclusion, the weak transformability of strain LMG18311 results from a low expression of *comR*, solely. This may prevent the activation of the positive feedback loop acting on ComS production.

### Increasing ComR concentration induces competence in the weakly transformable strain LMG18311

To further confirm the key role of ComR concentration on the transformability of strain LMG18311, we tested the consequence of *comR* overexpression on this phenotype. As expected, both the mean transformation frequency (4.4 × 10^−4^ ± 4.0 × 10^−4^ vs. 2.1 × 10^−6^ ± 5.0 × 10^−5^) and the mean maximum specific luciferase activity (RLU OD${}_{600}^{-1}$) of P_*comX*_-*luxAB* fusions (6.7 × 10^6^ ± 3.5 × 10^6^ vs. 4.4 × 10^4^ ± 1.2 × 10^3^) increased significantly in the reporter derivative strain carrying the *comR* overexpression vector compared to those carrying the empty vector. Interestingly, besides a high variability in the amplitude of P_*comX*_ activity (Figure 6C and data not shown), the various LMG18311 ComR^+^ clones also display variable (but reproducible) kinetics patterns, which is either monophasic (4/10 clones), biphasic (5/10 clones), or in rare instances triphasic (1/10 clone) (the specific luciferase activity of representative clones are presented in Figure 6C). To rationally interpret these results, we simulated increasing levels of ComR production by multiplying its maximum basal rate by a range of increasing values (1.5–20). For IF ranging from 3.25 to 7 (Figure 6D and data not shown), the time-course evolution of ComX concentration displays an oscillating behavior. The number of phases is directly proportional to the level of ComR production. At higher IF (Figure 6D), the phases merge while maximum amplitude continues to increase until saturation. Those predictions suggest that the different behaviors observed among LMG18311 ComR^+^ clones result from different levels of *comR* expression, which could for instance be due to different copy numbers of the corresponding expression vector.

## Discussion

Competence for natural transformation is a major driving force in bacterial evolution. Hence, it is of key importance to understand how this transient phenomenon takes place and is regulated. In Gram-positive bacteria, the best documented competence regulation systems are those of the model species *S. pneumoniae* (ComCDE-ComX relay system) (Claverys et al., 2006) and *Bacillus subtilis* (ComK master regulator) (Hamoen et al., 2003). During the last decade, the regulatory cascades governing competence in these two species have been mathematically modeled for helping to solve some important aspects such as the entry in the competence state (Leisner et al., 2009; Liebal et al., 2010), competence shut-off (Karlsson et al., 2007; Schultz et al., 2007), competence bistability (Maamar and Dubnau, 2005; Dubnau and Losick, 2006; Son et al., 2012; Xi et al., 2013), decision-making process between developmental programs (competence vs. sporulation) (Schultz et al., 2009), or benefits and cost of competence development (Moradigaravand and Engelstadter, 2013). These modeling approaches were very useful to decipher these processes and to obtain in some cases counter-intuitive explanations.

The ComRS-ComX relay system, which regulates competence in a majority of streptococcal groups, was recently discovered (for a review, see Fontaine et al., 2015). Even if the available experimental data begin to accumulate, a series of questions remain open on its functioning such as the topology of the regulatory cascade or the critical components of this system responsible for the on-off state of competence development. Although a simple mathematical model integrating ComRS was built to study environmental inputs on competence bistability in *S. mutans* (Son et al., 2012), a kinetic model that integrates all known steps of *comX* regulation by ComRS was missing until now.

### The ComRS dynamic model: limitations and future developments

At this stage, our model presents some limitations partially due to mechanistic or meta-hypotheses that were done to construct the model. First, the model neglects the putative cell-to-cell communication role of ComS. However, although “self”-communication dominates over “others”-communication in peptide diffusion-limiting conditions (such as modeled here), the combination of specific parameters may influence the output of social behaviors mediated by pheromone-based regulatory systems, such as the rate of peptide secretion and importation, the strength of the positive feedback loop (Youk and Lim, 2014) and/or the possibility of communication mediated by physical contacts between cells. In the future, both aspects (egoist and altruistic) of ComS signaling will be integrated in our model in order to study their relative contribution in competence regulation in various growth conditions.

Other limitations of our current model are that (i) the simulated cell growth is always assumed to be the same between WT and mutants while altered growth profiles were experimentally observe in mutant strains, (ii) the model has been calibrated and evaluated only for CDML growth conditions (iii) the model only considers the effect of proximal actors on *comX* expression while the effects of all distal regulators are summed up in the temporal gaussian term (*N*(*t*)) of ComR due to a lack of knowledge on distal regulation, and (iv) the model is not calibrated on the true abundance of ComR, ComS, and ComX proteins, which is unknown in ComRS-containing streptococci, but on expression data based on the activity of the luciferase reporter system. This last point implicates that the model remains qualitative and could be used to predict behaviors and to compare quantitative effects, but not to directly infer quantitative data. So, future improvements of the model will aim to solve some of these limitations such as integrating detailed distal regulations (transcriptional and post-transcriptional), improving quantitative predictions by measuring the time-course production of key players of the system during growth, or taking into account both RNA and protein levels.

Finally, the question of the portability of the model developed for *S. thermophilus* to other ComRS-containing streptococci is an important aspect. Although differences exist between species regarding for instance the diffusion properties of ComS and the mechanism of competence shut-off (see below), the topology of the ComX induction cascade as well as growth conditions allowing its activation (i.e., CDM conditions) are strongly conserved among ComRS-containing streptococci (for a review, see Fontaine et al., 2015). This suggests that the adaptation of the model to other phylogenetically close streptococci (e.g., *S. mutans, S. salivarius*) could be achieved without major adjustments.

### ComR abundance is critical for competence activation

One of the interesting outputs of the use of the model is that, besides the ComS pheromone, ComR is a limiting factor for maximum competence development of *S. thermophilus* in CDM conditions (Figure 5A). This was experimentally validated: the *comR* overexpression in strain LMD-9, which develops competence spontaneously, and strain LMG18311, which is weakly competent, resulted in a mean 10- and 100-fold higher level of *comX* expression, respectively (Figures 5C, 6C). It is noteworthy that increasing ComS abundance in strains LMD-9 and LMG18311 (through *in silico* or *in vivo* exogenous peptide supplementation) (Figures 6A,B) induces a similar positive effect on competence development but the amplitude of the response is correlated to the number of ComR molecules produced in each strain. Indeed, the induction of *comX* by saturating ComS concentrations remains stronger in strain LMD-9 vs. LMG18311 (Figures 6A,B). In regard to the topology of the competence induction cascade, ComR is indeed pivotal as its amount will determine the number of ComRS complexes which will be formed. A critical amount of ComRS complexes is probably required to activate the positive feedback loop acting on *comS* expression and hence induce competence in the population. Our results strongly suggest that this minimal amount is not reached in strain LMG18311 because of a too low *comR* expression level. The model predicts that increasing the ComR concentration in this strain shifts the balance toward formation of enough ComRS complexes to spontaneously induce competence. Interestingly, competence also turns on much earlier during growth of ComR^+^ vs. WT cells because of the immediate production of high amount of ComRS (data not shown), indicating that the timing device is constrained by the kinetics and amplitude of ComR production, solely. Such a critical role of ComR abundance does not seem to be restricted to the *S. thermophilus* species since it was previously reported that ComR overexpression in *S. mutans* UA159 resulted in a 10-fold increase in transformability (Mashburn-Warren et al., 2010). Increasing ComR production could thus provide a simple way to render weakly or non-competent strains highly competent without the requirement of adding synthetic ComS.

The role of ComR as a limiting factor of competence may be related to the structure of the ComRS signaling cascade in which ComR production is independent of the positive feedback loop (Figure 1). Indeed, this contrasts with the topology of most cell-cell communication systems in which productions of master regulator and pheromone are auto-regulated and coordinated at the transcriptional level, as for instance in the ComCDE system controlling competence in *S. pneumoniae* (Fontaine et al., 2015) and the streptococcal Rgg/Shp systems (Pérez-Pascual et al., 2015). This ensures that the same amount of regulator and associated pheromone are produced in these systems. The evolutionary consequence of uncoupling ComR and ComS production may be advantageous to fine-tune both kinetics and amplitude of competence activation in response to various environmental inputs. Currently, the modulation of *comR* transcription by distal regulatory systems (e.g., BsrRM-HdrRM relay and ScnRK) has only been shown in *S. mutans* (Okinaga et al., 2010; Xie et al., 2010; Kim et al., 2013). In addition, other signaling systems (i.e., *S. pyogenes* Rgg/Shp, and *S. pneumoniae* TprA/PhrA) of the RNPP family (the latter includes ComR) have also been shown as transcriptionally regulated by distal systems sensing either metal availability (Chang et al., 2015) or carbon source (Chang et al., 2015; Hoover et al., 2015). In *S. thermophilus*, no distal regulation acting on *comR* expression has been reported so far.

The comparative study of strains LMD-9 and LMG18311 indicate that competence heterogeneity between *S. thermophilus* strains (Fontaine et al., 2010b) could partially be due to differences in the *comR* expression level. The origin of these differences (i.e., LMD-9 vs. LMG18311) is not clear. Our model predicts that the activation or de-repression of *comR* expression by distal regulators, rather than the basal expression, is affected in strain LMG18311 compared to strain LMD-9 (Figure 6B). It may either be due to sequence polymorphism of the promoter region that affects binding of regulator(s), or to the presence of mutations in some *comR* distal regulators in poorly competent strains. Alternatively, in regard to the exceptional transformability of strain LMD-9 within the *S. thermophilus* species, it is possible that LMD-9 harbors specific mutations in *comR* regulator(s) allowing competence induction in otherwise non-permissive growth conditions. Consistent with this hypothesis of competence deregulation, strains of *Bacillus licheniformis* displaying efficient transformability carry mutations in competence regulator genes as a probable consequence of domestication (Jakobs and Meinhardt, 2015). Swapping *comR* promoter regions and determination of the sub-regions important for *comR* expression could help to begin answering this question.

### Insights into competence shut-off

In 2007, the use of an ODE mathematical model of the ComCDE system has suggested that the competence shut-off mechanism in *S. pneumoniae* was likely to involve a late phase repressor protein (Karlsson et al., 2007). A couple of years later, the shut-off mechanism in *S. pneumoniae* was experimentally unraveled and takes place at multiple levels: (i) competition between ComE and ComE~P (Martin et al., 2013), (ii) inhibition of ComX activity (Weng et al., 2013), and (iii) direct inhibition of ComE~P by DprA (Mirouze et al., 2013). The latter is a late phase protein that was initially proven as involved in DNA integration by recruiting RecA (Bergé et al., 2003; Mortier-Barrière et al., 2007).

In ComRS-containing streptococci, the shut-off mechanism is poorly understood and seems to differ among species (Fontaine et al., 2015). In *S. mutans*, it was shown that this mechanism was at least due to a post-translational control of ComX stability by the concerted action of the MecA adaptor protein and the ClpCP proteolytic system (Tian et al., 2013; Dong et al., 2014). In contrast, it has been shown that the MecA-ClpCP machinery does not contribute to the shut-off in *S. thermophilus* (Boutry et al., 2012; Wahl et al., 2014). However, it has been observed that ComX-negative strains exhibit a longer and stronger activation of genes belonging to the ComRS core regulon (i.e., *comS* and *comX*), without affecting *comR* expression (Boutry et al., 2013). This behavior could be simulated with our predictive model (Figures 4C,D), confirming that at least one late gene product is partially responsible for competence shut-off and acts by negatively affecting ComRS activity.

In our model, the feedback inhibition loop via the putative effector of shut-off ComZ is directed on the ComRS complex in order to form a ternary complex ComRSZ (Figure 1). By analogy with *S. pneumoniae*, the orthologue of DprA (which interacts and inhibits ComE activity in the pneumococcus) was an obvious putative candidate for this function. However, the early competence phase of *S. thermophilus* LMD-9 is not affected in a *dprA* deletion mutant compared to the WT (see Supplementary Figure S6), suggesting that the DprA orthologue is not involved in competence exit in this species. Although other late competence gene(s) could substitute DprA for sequestering active ComRS complexes in *S. thermophilus*, we cannot rule out alternative inactivation mechanisms such as (i) degradation of ComR and/or ComS, (ii) inactivation by post-translational modification of ComR and/or ComS (e.g., ComR phosphorylation), or (iii) inhibition of formation or activity of the ComRS complex (e.g., inhibitory peptide, competition for DNA binding). Changing the target of the negative loop in the model combined with modifications of parameters affecting the abundance of ComR and/or ComS could be useful to define at which level(s) the shut-off is taking place.

### Oscillatory behavior of competence activation

Besides the critical role of ComR in the strength of competence activation, ComR abundance also regulates an oscillatory behavior to competence activation. Indeed, an oscillating profile of *comX* expression was observed *in silico* and experimentally in the *comR*-overexpressing strain LMG18311 (Figures 6C,D). This provides an additional validation of the topological organization and the dynamical calibration of our model. Indeed, Novák and Tyson (2008) showed that the four minimal requirements for oscillations are: (i) a negative feedback loop, which is made in our setting of ComRS → ComX → ComZ⊣ComRS, (ii) a delay in this negative loop, implemented here by the positive loop made of ComS → ComS^ext^ → ComS^*^ → ComRS → ComS, (iii) sufficient non-linearity, which arises here from our non-linear activation/repression functions and from the mass action law of the ComRS assembly with *n* = 2.5, and (iv) appropriate time-scales of the opposite forces, confirming that our parameter set is coherent. Moreover, according to the notation of Lenz and Sogaard-Andersen (2011), our system can be classified as a relaxation oscillator due to its structure (a time-delayed negative feedback loop coupled to a positive feedback loop), hence operating in a broad frequency range. Indeed, both amplitude and frequency of oscillations can be modulated by varying the ComR production level (Figures 6C,D).

Temporal oscillations have been reported in a wide range of biological networks and organisms: from bacterial gene circuits (Schultz et al., 2009, 2013) to eukaryotic circadian rhythms (Lenz and Sogaard-Andersen, 2011) and quorum-sensing systems (Sgro et al., 2015). In addition, synthetic oscillators have been successfully built (Elowitz and Leibler, 2000). Interestingly, cycles in the competence state have already been reported for *S. pneumoniae* (Chen and Morrison, 1987) and *Helicobacter pylori* (Baltrus and Guillemin, 2006). In *S. pneumoniae*, the activation of these multiple competence cycles (up to three during growth in batch cultures) in a WT strain was observed at high initial pH values (>7.25) while competence is inhibited in acidic conditions (Chen and Morrison, 1987). In WT strains of *Helicobacter pylori*, multiple competence phases occurred spontaneously but appeared to be strain-dependent (Baltrus and Guillemin, 2006). Although a similar cycling behavior is observed here in an artificial genetic background of *S. thermophilus*, oscillation in competence gene activation was also seen in response to ComS addition in the closely related species *S. salivarius* (data not shown), indicating that competence oscillation could be an intrinsic property of the ComRS signaling system. It has been previously shown that oscillations are synchronized in the whole population through quorum-sensing inducing molecules (McMillen et al., 2002; Schwab et al., 2012; Singh and Parmananda, 2013). However, it remains to be determined here if oscillations are taking place at the whole population level or if successive subpopulations are activating a single competence episode. Future time-lapse experiments at the single cell level with a GFP-reporter strain of competence activation will be needed to answer this question.

Finally, the question of the biological advantage conferred by an oscillating competence state has not yet been addressed. We can speculate that the benefit could be the lengthening of the transformation process in conditions where competence is highly beneficial for the population. Oscillations would then allow recovery phases that could be needed to relieve the inhibition of cell division resulting from competence activation, hence implementing a compromise between competence and growth, two processes that could be mutually exclusive (Haijema et al., 2001; Qi et al., 2005). However, one must keep in mind that the current observations were obtained in artificial laboratory conditions in which bacteria could exhibit exotic behaviors that would not or rarely be seen in natural conditions.

## Concluding remarks

To conclude, we designed an original approach combining mathematical modeling with molecular biology tools to build a robust data-based model of the ComRS signaling system, which helped us to confirm the topology of the competence regulatory network and to identify its critical components in salivarius streptococci. We demonstrated the key hierarchical importance of ComR in transformation efficiency and timing of competence activation, compatible with its putative role of master integration-sensor of distal regulations by environmental signals. In addition, our model shed light on the competence shut-off mechanism and proposed that the topology of the regulatory network based on ComRS allows an oscillatory behavior of competence activation. Future work will consist in exploiting our model to identify more finely at which level the shut-off is taking place in order to identify putative key player(s) involved in this mechanism. A possible oscillating competence state is also an exciting aspect that will require further investigation such as its occurrence at the single cell level and its biological relevance.

### Conflict of interest statement

The authors declare that the research was conducted in the absence of any commercial or financial relationships that could be construed as a potential conflict of interest.
