# Supplementary material for: Modeling of the ComRS Signaling Pathway Reveals the Limiting Factors Controlling Competence in Streptococcus thermophilus
Source: Front Microbiol. 2015 Dec 22;6:1413. doi: 10.3389/fmicb.2015.01413 (PMC4686606; doi:10.3389/fmicb.2015.01413)
Supplement: Supplementary file 1 [file DataSheet1.DOCX]

function ODE_model()

%%

% To run our model, please copy-paste this text in a matlab file named

% 'ODE_model.m'

%

% This code contains :

% the model calibration (all parameters definitions),

% the model validation,

% and the various outputs of the model.

%

% It takes approximately 30 seconds to run.

% In order to speed up, please uncomment the unneeded parts.

%

% If you have any question, feel free to contact Mrs Laurie Haustenne :

% laurie.haustenne@uclouvain.be

%

clc

clear all

close all

%% GROWTH (assumed to be the same for all mutants)

disp('Processing growth...')

t = (0:10:360)'; % Time in minutes : from 1 to 360 minutes

OD_600 = xlsread('Experimental_data.xlsx','Growth rate','B4:B40'); % Growth curve (OD600)

Xm = OD_600*5*10^8; % Xm is now in [cell/mL]

subplot(3,2,1)

plot(t,Xm,'b*')

X_ = @(x) (x).^5./(200^5+(x).^5).*(Xm(end)) + Xm(1);

hold on

plot(t,X_(t),'-g')

title('Growth [cells/mL]')

mu_ = @(x) (Xm(end)*(5.*(x).^4.*(200^5+(x).^5)-(x).^5.*5.*(x).^4)./((200^5+(x).^5).^2))./(((x).^5.*Xm(end))./(200^5+(x).^5) + Xm(1));

% mu = dX/dt * 1/X(t) : ( f'g - fg' )/ g^2 *1/X

subplot(3,2,2)

plot(t,mu_(t),'-g'); % We see that the maximum division time is 30 min

title('Growth rate [1/min]')

function result = integ(vec)

result = zeros(1,length(vec));

for i=1:length(vec)

result(i) = quad(mu_,vec(1),vec(i));

end

end

X_approx = Xm(1)*exp(integ(t));

subplot(3,2,1)

plot(t,X_approx,'r.')

disp(' ...done')

%% PRODUCTION RATES

disp('Computing the production rates...')

% Reading the RLU curves

RLU_comS = xlsread('Experimental_data.xlsx','RLU','S4:S40'); % PcomS in [RLU]

RLU_comX = xlsread('Experimental_data.xlsx','RLU','T4:T40'); % PcomX in [RLU]

RLU_comR = xlsread('Experimental_data.xlsx','RLU','U4:U40'); % PcomR in [RLU]

subplot(3,2,3)

plot(t,RLU_comS,'b*')

hold on

title('PcomS and PcomX (in red) [RLU]')

subplot(3,2,4)

plot(t,RLU_comR,'g*')

title('PcomR [RLU]')

subplot(3,2,3)

plot(t,RLU_comX,'r*')

RLU_comS = RLU_comS./X_(t); % PcomS in [RLU/cells]

RLU_comR = RLU_comR./X_(t); % PcomR in [RLU/cells]

RLU_comX = RLU_comX./X_(t); % PcomX in [RLU/cells]

% Approximation of these curves by skewed gaussians

H1 = 4.4;

ep1 = 108;

w1 = 45;

alpha1 = 4;

RLU_comS_ = @(x) H1*(1/(w1*sqrt(2*pi)))*exp((-((x-ep1)/w1).^2)./2).*(1+erf(alpha1*((x-ep1)./w1)./(sqrt(2))));

H2 = 0.17;

ep2 = 70;

w2 = 60;

alpha2 = 2;

H2bis = 0.005;

ep2bis = 180;

w2bis = 20;

alpha2bis = 4;

RLU_comR_ = @(x) H2*(1/(w2*sqrt(2*pi)))*exp((-((x-ep2)/w2).^2)./2).*(1+erf(alpha2*((x-ep2)./w2)./(sqrt(2))))...

+ H2bis*(1/(w2bis*sqrt(2*pi)))*exp((-((x-ep2bis)/w2bis).^2)./2).*(1+erf(alpha2bis*((x-ep2bis)./w2bis)./(sqrt(2))));

H3 = 0.8;

ep3 = 130;

w3 = 45;

alpha3 = 5;

RLU_comX_ = @(x) H3*(1/(w3*sqrt(2*pi)))*exp((-((x-ep3)/w3).^2)./2).*(1+erf(alpha3*((x-ep3)./w3)./(sqrt(2))));

subplot(3,2,5)

plot(t,RLU_comS,'b*',t,RLU_comS_(t),'-b')

hold on

title('PcomS and PcomX [RLU/cell]')

subplot(3,2,6)

plot(t,RLU_comR,'g*',t,RLU_comR_(t),'-g') % ------------ units : [RLU / CELL] ----------------

title('PcomR [RLU/cell]')

subplot(3,2,5)

plot(t,RLU_comX,'r*',t,RLU_comX_(t),'-r')

% Computation of the production rates

d_lux = log(2)/45; % T 1/2 = 45' for luciferase

% derivative : dP/dt in [RLU/cell*min]

derS_ = @(x) diff(RLU_comS_(x))./diff(x);

derS = [derS_(t(1:2)); derS_(t(:))];

figure

subplot(3,3,1)

plot(t,derS,'-g',t,zeros(size(t)),'--r')

hold on

plot(t,d_lux*RLU_comS_(t),'k')

hold on

plot(t,mu_(t).*RLU_comS_(t),'b')

hold on

prodS = derS + d_lux*RLU_comS_(t) + mu_(t).*RLU_comS_(t); % ------------ units : [RLU / CELL*MIN] ----------------

plot(t,prodS,'r') % (because the factor 1/k is not yet introduced here (RLUsurMOL))

title('Raw ProdS [RLU / CELL*MIN]')

derR_ = @(x) diff(RLU_comR_(x))./diff(x);

derR = [derR_(t(1:2)); derR_(t(:))];

subplot(3,3,2)

plot(t,derR,'-g',t,zeros(size(t)),'--r')

hold on

plot(t,d_lux*RLU_comR_(t),'k')

hold on

plot(t,mu_(t).*RLU_comR_(t),'b')

hold on

prodR = derR + d_lux*RLU_comR_(t) + mu_(t).*RLU_comR_(t);

plot(t,prodR,'r')

title('Raw ProdR [RLU / CELL*MIN]')

derX_ = @(x) diff(RLU_comX_(x))./diff(x);

derX = [derX_(t(1:2)); derX_(t(:))];

subplot(3,3,3)

plot(t,derX,'-g',t,zeros(size(t)),'--r')

hold on

plot(t,d_lux*RLU_comX_(t),'k')

hold on

plot(t,mu_(t).*RLU_comX_(t),'b')

hold on

prodX = derX + d_lux*RLU_comX_(t) + mu_(t).*RLU_comX_(t);

plot(t,prodX,'r')

title('Raw ProdX [RLU / CELL*MIN]')

% Smoothing the negative part (negative production is impossible).

% To do this, we approximate the curves by new skewed gaussians.

HS = 0.19;

epS = 103;

wS = 25;

alphaS = 2;

prodSplus_ = @(x) HS*(1/(wS*sqrt(2*pi)))*exp((-((x-epS)/wS).^2)./2).*(1+erf(alphaS*((x-epS)./wS)./(sqrt(2))));

subplot(3,3,4)

plot(t,prodS,'r',t,prodSplus_(t),'b')

title('Smoothed prodS [RLU / CELL*MIN]')

HR = 0.0062;

epR = 60;

wR = 55;

alphaR = 2;

prodRplus_ = @(x) HR*(1/(wR*sqrt(2*pi)))*exp((-((x-epR)/wR).^2)./2).*(1+erf(alphaR*((x-epR)./wR)./(sqrt(2))));

subplot(3,3,5)

plot(t,prodR,'r',t,prodRplus_(t),'b') % ------------ units : [RLU / CELL*MIN] ----------------

title('Smoothed prodR [RLU / CELL*MIN]') % The units remain the same, it is just an approximation

HX = 0.0335;

epX = 126;

wX = 20;

alphaX = 2;

prodXplus_ = @(x) HX*(1/(wX*sqrt(2*pi)))*exp((-((x-epX)/wX).^2)./2).*(1+erf(alphaX*((x-epX)./wX)./(sqrt(2))));

subplot(3,3,6)

plot(t,prodX,'r',t,prodXplus_(t),'b')

title('Smoothed prodX [RLU / CELL*MIN]')

% Scaling

b1 = max(prodSplus_(t));

d1 = 20; % Max speed : 1 ComS in 3sec : 20 ComS/minute

RLUsurMOL = b1/d1;

% Final production curves

ProdS_ = @(x) (1/RLUsurMOL)*HS*(1/(wS*sqrt(2*pi)))*exp((-((x-epS)/wS).^2)./2).*(1+erf(alphaS*((x-epS)./wS)./(sqrt(2))));

subplot(3,3,7)

plot(t,ProdS_(t))

title('Scaled prodS [MOL / CELL*MIN]')

ProdR_ = @(x) (1/RLUsurMOL)*HR*(1/(wR*sqrt(2*pi)))*exp((-((x-epR)/wR).^2)./2).*(1+erf(alphaR*((x-epR)./wR)./(sqrt(2))));

subplot(3,3,8)

plot(t,ProdR_(t)) % ------------ units : [MOL / CELL*MIN] ----------------

title('Scaled prodR [MOL / CELL*MIN]') % Here we have converted the RLU in #molecules (through 1/RLUsurMOL)

ProdX_ = @(x) (1/RLUsurMOL)*HX*(1/(wX*sqrt(2*pi)))*exp((-((x-epX)/wX).^2)./2).*(1+erf(alphaX*((x-epX)./wX)./(sqrt(2))));

subplot(3,3,9)

plot(t,ProdX_(t))

title('Scaled prodX [MOL / CELL*MIN]')

disp(' ...done')

%% PARAMETERS OF S2 (CONSTRAINED SYSTEM)

rout = 1; % Exportation rate constant

rin = 0.01; % Importation rate constant

n = 2.5; % Hill coefficient (Son, 2012)

c_RS = 0.1; % Complex formation rate constant

vmaxZ = 1; % Maximal activation rate of ComZ by ComX

nZ = 3; % Hill coefficient

KZ = 15; % Required concentration of ComX for half-maximum synthesis rate of ComZ

vmaxRSZ = 0.04; % Maximal inactivation rate of ComRS by ComZ

KRSZ = 0.5; % Required concentration of ComRS*ComZ for half-maximum inactivation rate of ComRS

dComSext = 0.07; % Degradation rate constant of ComSext

dComSstar = 0.07; % Degradation rate constant of ComSstar

dComR = 0.01; % Degradation rate constant of ComR

dComRS = 0.01; % Degradation rate constant of ComRS

dX = 0.2; % Degradation rate constant of ComX (Karlsson, 2007)

dComZ = 0.01; % Degradation rate constant of ComZ

%% PARAMETERS OF S1 (FULL SYSTEM, 9 parameters more)

VmaxS = 4; % Maximal activation rate of ComS by ComRS

KS = 0.5; % Required concentration of ComRS for half-maximum synthesis rate of ComS

centre = 90; % Center of the gaussian activation of ComR (noted as 'm' in the paper)

sigma = 35; % Standard deviation of the activation of ComR

A = 27; % Amplitude of the gaussian activation of ComR

VmaxX = 5.3; % Maximal activation rate of ComX by ComRS

KX = 1; % Required concentration of ComRS for half-maximum synthesis rate of ComX

%% Optional parameters

% These parameters are used for later simulations :

BS = 1; BX = 1; BR = 1;

VmaxR = 0.4; KR = 1.5; nR = 1; % To test the hypothesis that ComR is in the positive feedback loop

%% --------------- S2 CONSTRAINED SYSTEM --------------------

% THIS SYSTEM ONLY CORRESPONDS TO A WT, "NORMAL" SITUATION, WITHOUT ADDED

% COMS (TO BE COHERENT WITH EXPERIMENTAL PRODUCTIONS)

function result = S2_constrained_system(t,y)

dComSdt = ProdS_(t) - rout*y(1) - mu_(t)*y(1);

dComSextdt = rout*y(1) - rin*y(2) - mu_(t)*y(2) - dComSext*y(2);

dComSstardt = rin*y(2) - mu_(t)*y(3) - c_RS*(y(3)*y(4))^n - dComSstar*y(3);

dComRdt = ProdR_(t) - mu_(t)*y(4) - c_RS*(y(3)*y(4))^n - dComR*y(4);

dComRSdt = (1/2)*c_RS*(y(3)*y(4))^n - mu_(t)*y(5) - vmaxRSZ*((y(5)*y(7))/(KRSZ + y(5)*y(7))) - dComRS*y(5);

dComXdt = ProdX_(t) - mu_(t)*y(6) - dX*y(6);

dComZdt = vmaxZ*((y(6)^nZ)/(KZ^nZ + y(6)^nZ)) - mu_(t)*y(7)- vmaxRSZ*((y(5)*y(7))/(KRSZ + y(5)*y(7))) - dComZ*y(7);

result = [dComSdt; dComSextdt; dComSstardt; dComRdt; dComRSdt; dComXdt; dComZdt];

end

%% INTEGRATION OF S2

disp('Integrating the S2 constrained system...')

sol1 = ode23(@S2_constrained_system, [t(1) t(end)], zeros(1,7));

figure

subplot(2,4,1)

plot(sol1.x,X_(sol1.x),'-g')

title('Growth [cells/mL]')

subplot(2,4,2)

plot(sol1.x, sol1.y(1,:),'-k')

hold on

title('ComS(t)')

subplot(2,4,3)

plot(sol1.x, sol1.y(2,:),'-k')

title('ComSext(t)')

hold on

subplot(2,4,4)

plot(sol1.x, sol1.y(3,:),'-k')

title('ComS*(t)')

hold on

subplot(2,4,5)

plot(sol1.x, sol1.y(4,:),'-k')

title('ComR(t)')

hold on

subplot(2,4,6)

plot(sol1.x, sol1.y(5,:),'-k')

title('ComRS(t)')

hold on

subplot(2,4,7)

plot(sol1.x, sol1.y(6,:),'-k')

title('ComX(t)')

hold on

subplot(2,4,8)

plot(sol1.x, sol1.y(7,:),'-k')

title('ComZ(t)')

hold on

disp(' ...done')

%%%%%%%%%%%%%%%%%%%%%%%%%%%%%%%%%%%%%%%%%%%%%%%%%%%%%%%%%%%%%%%%%%%%%%%%%%%%%%%%%%%%%%%%%%%%%%%%%%%%%%%%%%%%%%%%%%%%%%%%%%%%%%%%%%%%%%%%%%%%%%%%

%% BACK MODELING

disp('Back modeling : computing basal rates...')

% ------------- Basal rates based on RLU(t) -----------------------

% DPS_DeltaComR : no ComR => no feedback => approx of bs(t)

% Two clones tested :

bS_expe_1 = xlsread('Experimental_data.xlsx','bS','C3:C29');

bS_expe_2 = xlsread('Experimental_data.xlsx','bS','D3:D29');

bS_expe = (bS_expe_1 + bS_expe_2)/2;

ttt = linspace(0,263,27)'; % Time (263 minutes)

figure

subplot(2,2,1)

plot(ttt,bS_expe,'b*')

hold on

title('bS expe (t)[RLU]')

bS_expe = bS_expe./X_(ttt); % bS(t) in [RLU/cells]

subplot(2,2,2)

plot(ttt,bS_expe,'b*')

hold on

H1 = 0.85*10^(-2);

ep1 = 40;

w1 = 55;

alpha1 = 1.8;

bS_expe_ = @(x) H1*(1/(w1*sqrt(2*pi)))*exp((-((x-ep1)/w1).^2)./2).*(1+erf(alpha1*((x-ep1)./w1)./(sqrt(2))));

% Now, bS_expe is good for all t in [0 360], since the skewed gaussian

% "extends" the measurement between 0 and 263 minutes...

subplot(2,2,2)

plot(ttt,bS_expe_(ttt),'r')

title('bS(t) expe and its approximation [RLU/cell]')

hold on

% Computation of the corresponding production in molecules/cell*min

der_ = @(x) diff(bS_expe_(x))./diff(x);

der = [der_(t(1:2)); der_(t(:))];

subplot(2,2,3)

plot(t,der,'-g',t,zeros(size(t)),'--r')

hold on

plot(t,d_lux*bS_expe_(t),'k')

hold on

plot(t,mu_(t).*bS_expe_(t),'b')

hold on

prod_bS = der + d_lux*bS_expe_(t) + mu_(t).*bS_expe_(t);

plot(t,prod_bS,'r')

title('Raw prod bS [RLU / CELL*MIN]')

subplot(2,2,4)

plot(t,(1/RLUsurMOL)*prod_bS,'r');

hold on

H1 = 0.26*10^(-3);

ep1 = 20;

w1 = 55;

alpha1 = 1.8;

approx_bS_ = @(x) 1*(1/RLUsurMOL)*H1*(1/(w1*sqrt(2*pi)))*exp((-((x-ep1)/w1).^2)./2).*(1+erf(alpha1*((x-ep1)./w1)./(sqrt(2))));

b_ = approx_bS_;

bS_ = @(x) (1/RLUsurMOL)*H1*(1/(w1*sqrt(2*pi)))*exp((-((x-ep1)/w1).^2)./2).*(1+erf(alpha1*((x-ep1)./w1)./(sqrt(2))));

bX_ = @(x) (1/RLUsurMOL)*H1*(1/(w1*sqrt(2*pi)))*exp((-((x-ep1)/w1).^2)./2).*(1+erf(alpha1*((x-ep1)./w1)./(sqrt(2))));

bR_ = @(x) (1/RLUsurMOL)*H1*(1/(w1*sqrt(2*pi)))*exp((-((x-ep1)/w1).^2)./2).*(1+erf(alpha1*((x-ep1)./w1)./(sqrt(2))));

plot(t,b_(t),'c--')

title('Basal rate [molecules/CELL*MIN] : based on RLU')

disp(' ...done')

disp('Computing the inverse productions...')

% based on RLU DPS Delta_ComR (based on y1)

ProdS_inverse = bS_(sol1.x) + VmaxS*(sol1.y(5,:)/(KS+sol1.y(5,:)));

ProdR_inverse = bR_(sol1.x) + (A/(sigma*sqrt(2*pi)))*exp(-0.5*((sol1.x-centre)./sigma).^2); % our working hypothesis

ProdR_inverseComRS = bR_(sol1.x) + VmaxR*((sol1.y(5,:).^nR)./(KR^nR+sol1.y(5,:).^nR)); % false hypothesis

ProdX_inverse = bX_(sol1.x) + VmaxX*(sol1.y(5,:)./(KX+sol1.y(5,:)));

figure

subplot(1,4,1)

plot(t,ProdS_(t), sol1.x,ProdS_inverse,'r');

title('Raw iprodS')

subplot(1,4,2)

plot(t,ProdR_(t), sol1.x,ProdR_inverse,'r');

title('Raw iprodR : true hyp')

subplot(1,4,3)

plot(t,ProdR_(t), sol1.x,ProdR_inverseComRS,'r');

title('Raw iprodR : false hyp')

subplot(1,4,4)

plot(t,ProdX_(t), sol1.x,ProdX_inverse,'r');

title('Raw iprodX')

disp(' ...done')

%% --------------- S1 FULL SYSTEM --------------------

function result = system_S1(t,y)

dComSdt = BS*bS_(t) + VmaxS*((y(5))/(KS+y(5))) - rout*y(1) - mu_(t)*y(1);

dComSextdt = rout*y(1) - rin*y(2) - mu_(t)*y(2) - dComSext*y(2);

dComSstardt = rin*y(2) - mu_(t)*y(3) - c_RS*(y(3)*y(4))^n - dComSstar*y(3);

dComRdt = BR*bR_(t) + (A/(sigma*sqrt(2*pi)))*exp(-0.5*((t-centre)/sigma)^2) - mu_(t)*y(4) - c_RS*(y(3)*y(4))^n - dComR*y(4);

dComRSdt = (1/2)*c_RS*(y(3)*y(4))^n - mu_(t)*y(5) - vmaxRSZ*((y(5)*y(7))/(KRSZ + y(5)*y(7))) - dComRS*y(5);

dComXdt = BX*bX_(t) + VmaxX*((y(5))/(KX+y(5))) - mu_(t)*y(6) - dX*y(6);

dComZdt = vmaxZ*((y(6)^nZ)/(KZ^nZ + y(6)^nZ)) - mu_(t)*y(7) - vmaxRSZ*((y(5)*y(7))/(KRSZ + y(5)*y(7))) - dComZ*y(7);

result = [dComSdt; dComSextdt; dComSstardt; dComRdt; dComRSdt; dComXdt; dComZdt];

end

%% -------------- INTEGRATION ----------------

disp('Integrating the S1 full system...')

sol2 = ode23(@system_S1, [t(1) 410], zeros(1,7));

figure

subplot(2,4,1)

plot(sol2.x,X_(sol2.x),'-g')

title('Growth [cells/mL]')

subplot(2,4,2)

plot(sol2.x, sol2.y(1,:),'-k')

hold on

title('ComS(t)')

subplot(2,4,3)

plot(sol2.x, sol2.y(2,:),'-k')

title('ComSext(t)')

hold on

subplot(2,4,4)

plot(sol2.x, sol2.y(3,:),'-k')

title('ComS*(t)')

hold on

subplot(2,4,5)

plot(sol2.x, sol2.y(4,:),'-k')

title('ComR(t)')

hold on

subplot(2,4,6)

plot(sol2.x, sol2.y(5,:),'-k')

title('ComRS(t)')

hold on

subplot(2,4,7)

plot(sol2.x, sol2.y(6,:),'-k')

title('ComX(t)')

hold on

subplot(2,4,8)

plot(sol2.x, sol2.y(7,:),'-k')

title('ComZ(t)')

hold on

disp(' ...done')

%% Simulation of a ComS- mutant

% No ComS can be produced

function result = KO_ComS(t,y)

dComSdt = 0;

dComSextdt = rout*y(1) - rin*y(2) - mu_(t)*y(2) - dComSext*y(2);

dComSstardt = rin*y(2) - mu_(t)*y(3) - c_RS*(y(3)*y(4))^n - dComSstar*y(3);

dComRdt = BR*bR_(t) + (A/(sigma*sqrt(2*pi)))*exp(-0.5*((t-centre)/sigma)^2) - mu_(t)*y(4) - c_RS*(y(3)*y(4))^n - dComR*y(4);

dComRSdt = (1/2)*c_RS*(y(3)*y(4))^n - mu_(t)*y(5) - vmaxRSZ*((y(5)*y(7))/(KRSZ + y(5)*y(7))) - dComRS*y(5);

dComXdt = BX*bX_(t) + VmaxX*((y(5))/(KX+y(5))) - mu_(t)*y(6) - dX*y(6);

dComZdt = vmaxZ*((y(6)^nZ)/(KZ^nZ + y(6)^nZ)) - mu_(t)*y(7) - vmaxRSZ*((y(5)*y(7))/(KRSZ + y(5)*y(7))) - dComZ*y(7);

result = [dComSdt; dComSextdt; dComSstardt; dComRdt; dComRSdt; dComXdt; dComZdt];

end

%% Simulation of a ComX- mutant

% No ComX can be produced

function result = KO_ComX(t,y)

dComSdt = BS*bS_(t) + VmaxS*((y(5))/(KS+y(5))) - rout*y(1) - mu_(t)*y(1);

dComSextdt = rout*y(1) - rin*y(2) - mu_(t)*y(2) - dComSext*y(2);

dComSstardt = rin*y(2) - mu_(t)*y(3) - c_RS*(y(3)*y(4))^n - dComSstar*y(3);

dComRdt = BR*bR_(t) + (A/(sigma*sqrt(2*pi)))*exp(-0.5*((t-centre)/sigma)^2) - mu_(t)*y(4) - c_RS*(y(3)*y(4))^n - dComR*y(4);

dComRSdt = (1/2)*c_RS*(y(3)*y(4))^n - mu_(t)*y(5) - vmaxRSZ*((y(5)*y(7))/(KRSZ + y(5)*y(7))) - dComRS*y(5);

dComXdt = 0;

dComZdt = vmaxZ*((y(6)^nZ)/(KZ^nZ + y(6)^nZ)) - mu_(t)*y(7) - vmaxRSZ*((y(5)*y(7))/(KRSZ + y(5)*y(7))) - dComZ*y(7);

result = [dComSdt; dComSextdt; dComSstardt; dComRdt; dComRSdt; dComXdt; dComZdt];

end

%% Simulation of a ComZ- mutant

% No ComZ can be produced

function result = KO_ComZ(t,y)

dComSdt = BS*bS_(t) + VmaxS*((y(5))/(KS+y(5))) - rout*y(1) - mu_(t)*y(1);

dComSextdt = rout*y(1) - rin*y(2) - mu_(t)*y(2) - dComSext*y(2);

dComSstardt = rin*y(2) - mu_(t)*y(3) - c_RS*(y(3)*y(4))^n - dComSstar*y(3);

dComRdt = BR*bR_(t) + (A/(sigma*sqrt(2*pi)))*exp(-0.5*((t-centre)/sigma)^2) - mu_(t)*y(4) - c_RS*(y(3)*y(4))^n - dComR*y(4);

dComRSdt = (1/2)*c_RS*(y(3)*y(4))^n - mu_(t)*y(5) - vmaxRSZ*((y(5)*y(7))/(KRSZ + y(5)*y(7))) - dComRS*y(5);

dComXdt = BX*bX_(t) + VmaxX*((y(5))/(KX+y(5))) - mu_(t)*y(6) - dX*y(6);

dComZdt = 0;

result = [dComSdt; dComSextdt; dComSstardt; dComRdt; dComRSdt; dComXdt; dComZdt];

end

%% Simulation of a ComR- mutant

% No ComR can be produced

function result = KO_ComR(t,y)

dComSdt = BS*bS_(t) + VmaxS*((y(5))/(KS+y(5))) - rout*y(1) - mu_(t)*y(1);

dComSextdt = rout*y(1) - rin*y(2) - mu_(t)*y(2) - dComSext*y(2);

dComSstardt = rin*y(2) - mu_(t)*y(3) - c_RS*(y(3)*y(4))^n - dComSstar*y(3);

dComRdt = 0;

dComRSdt = (1/2)*c_RS*(y(3)*y(4))^n - mu_(t)*y(5) - vmaxRSZ*((y(5)*y(7))/(KRSZ + y(5)*y(7))) - dComRS*y(5);

dComXdt = BX*bX_(t) + VmaxX*((y(5))/(KX+y(5))) - mu_(t)*y(6) - dX*y(6);

dComZdt = vmaxZ*((y(6)^nZ)/(KZ^nZ + y(6)^nZ)) - mu_(t)*y(7) - vmaxRSZ*((y(5)*y(7))/(KRSZ + y(5)*y(7))) - dComZ*y(7);

result = [dComSdt; dComSextdt; dComSstardt; dComRdt; dComRSdt; dComXdt; dComZdt];

end

%% Simulation of a ComR+ mutant

global R

R = 0.012*50;

function result = OVER_ComR(t,y)

dComSdt = BS*bS_(t) + VmaxS*((y(5))/(KS+y(5))) - rout*y(1) - mu_(t)*y(1);

dComSextdt = rout*y(1) - rin*y(2) - mu_(t)*y(2) - dComSext*y(2);

dComSstardt = rin*y(2) - mu_(t)*y(3) - c_RS*(y(3)*y(4))^n - dComSstar*y(3);

dComRdt = R + (A/(sigma*sqrt(2*pi)))*exp(-0.5*((t-centre)/sigma)^2) - mu_(t)*y(4) - c_RS*(y(3)*y(4))^n - dComR*y(4);

dComRSdt = (1/2)*c_RS*(y(3)*y(4))^n - mu_(t)*y(5) - vmaxRSZ*((y(5)*y(7))/(KRSZ + y(5)*y(7))) - dComRS*y(5);

dComXdt = BX*bX_(t) + VmaxX*((y(5))/(KX+y(5))) - mu_(t)*y(6) - dX*y(6);

dComZdt = vmaxZ*((y(6)^nZ)/(KZ^nZ + y(6)^nZ)) - mu_(t)*y(7) - vmaxRSZ*((y(5)*y(7))/(KRSZ + y(5)*y(7))) - dComZ*y(7);

result = [dComSdt; dComSextdt; dComSstardt; dComRdt; dComRSdt; dComXdt; dComZdt];

end

%% Simulation of a ComS+ mutant

global S

S = 0.012*50;

function result = OVER_ComS(t,y)

dComSdt = S + VmaxS*((y(5))/(KS+y(5))) - rout*y(1) - mu_(t)*y(1);

dComSextdt = rout*y(1) - rin*y(2) - mu_(t)*y(2) - dComSext*y(2);

dComSstardt = rin*y(2) - mu_(t)*y(3) - c_RS*(y(3)*y(4))^n - dComSstar*y(3);

dComRdt = BR*bR_(t) + (A/(sigma*sqrt(2*pi)))*exp(-0.5*((t-centre)/sigma)^2) - mu_(t)*y(4) - c_RS*(y(3)*y(4))^n - dComR*y(4);

dComRSdt = (1/2)*c_RS*(y(3)*y(4))^n - mu_(t)*y(5) - vmaxRSZ*((y(5)*y(7))/(KRSZ + y(5)*y(7))) - dComRS*y(5);

dComXdt = BX*bX_(t) + VmaxX*((y(5))/(KX+y(5))) - mu_(t)*y(6) - dX*y(6);

dComZdt = vmaxZ*((y(6)^nZ)/(KZ^nZ + y(6)^nZ)) - mu_(t)*y(7) - vmaxRSZ*((y(5)*y(7))/(KRSZ + y(5)*y(7))) - dComZ*y(7);

result = [dComSdt; dComSextdt; dComSstardt; dComRdt; dComRSdt; dComXdt; dComZdt];

end

%% KO ComS simulation

sol2 = ode23(@KO_ComS, [t(1) 410], zeros(1,7));

figure

subplot(2,4,1)

plot(sol2.x,X_(sol2.x),'-g')

title('Growth [cells/mL]')

subplot(2,4,2)

plot(sol2.x, sol2.y(1,:),'-r')

hold on

title('ComS(t)')

subplot(2,4,3)

plot(sol2.x, sol2.y(2,:),'-r')

title('ComSext(t)')

hold on

subplot(2,4,4)

plot(sol2.x, sol2.y(3,:),'-r')

title('ComS*(t)')

hold on

subplot(2,4,5)

plot(sol2.x, sol2.y(4,:),'-r')

title('ComR(t)')

hold on

subplot(2,4,6)

plot(sol2.x, sol2.y(5,:),'-r')

title('ComRS(t)')

hold on

subplot(2,4,7)

plot(sol2.x, sol2.y(6,:),'-r')

title('ComX(t)')

hold on

subplot(2,4,8)

plot(sol2.x, sol2.y(7,:),'-r')

title('ComZ(t)')

hold on

%% KO ComX

sol2 = ode23(@KO_ComX, [t(1) 410], zeros(1,7));

figure

subplot(2,4,1)

plot(sol2.x,X_(sol2.x),'-g')

title('Growth [cells/mL]')

subplot(2,4,2)

plot(sol2.x, sol2.y(1,:),'-r')

hold on

title('ComS(t)')

subplot(2,4,3)

plot(sol2.x, sol2.y(2,:),'-r')

title('ComSext(t)')

hold on

subplot(2,4,4)

plot(sol2.x, sol2.y(3,:),'-r')

title('ComS*(t)')

hold on

subplot(2,4,5)

plot(sol2.x, sol2.y(4,:),'-r')

title('ComR(t)')

hold on

subplot(2,4,6)

plot(sol2.x, sol2.y(5,:),'-r')

title('ComRS(t)')

hold on

subplot(2,4,7)

plot(sol2.x, sol2.y(6,:),'-r')

title('ComX(t)')

hold on

subplot(2,4,8)

plot(sol2.x, sol2.y(7,:),'-r')

title('ComZ(t)')

%% Overexpression of ComR

sol2 = ode23(@OVER_ComR, [t(1) 410], zeros(1,7));

figure

subplot(2,4,1)

plot(sol2.x,X_(sol2.x),'-g')

title('Growth [cells/mL]')

subplot(2,4,2)

plot(sol2.x, sol2.y(1,:),'-r')

hold on

title('ComS(t)')

subplot(2,4,3)

plot(sol2.x, sol2.y(2,:),'-r')

title('ComSext(t)')

hold on

subplot(2,4,4)

plot(sol2.x, sol2.y(3,:),'-r')

title('ComS*(t)')

hold on

subplot(2,4,5)

plot(sol2.x, sol2.y(4,:),'-r')

title('ComR(t)')

hold on

subplot(2,4,6)

plot(sol2.x, sol2.y(5,:),'-r')

title('ComRS(t)')

hold on

subplot(2,4,7)

plot(sol2.x, sol2.y(6,:),'-r')

title('ComX(t)')

hold on

subplot(2,4,8)

plot(sol2.x, sol2.y(7,:),'-r')

title('ComZ(t)')

%% Dose-responses to ComS peptide

disp('Simulating dose-responses...')

a = [0 1 2 5 10 20 50 100 200 500 1000 2000];

mamax = zeros(1, length(a));

for h=1:length(a)

solh = ode23(@system_S1, [t(1) t(end)], [0,a(h),0,0,0,0,0]);

maxou = max(solh.y(6,:));

mamax(h) = maxou;

end

figure

plot(a, mamax,'g')

hold on

disp(' ...WT : done')

for h=1:length(a)

solh = ode23(@KO_ComS, [t(1) t(end)], [0,a(h),0,0,0,0,0]);

maxou = max(solh.y(6,:));

mamax(h) = maxou;

end

plot(a, mamax,'k')

hold on

disp(' ...KO ComS : done')

for h=1:length(a)

solh = ode23(@OVER_ComR, [t(1) t(end)], [0,a(h),0,0,0,0,0]);

maxou = max(solh.y(6,:));

mamax(h) = maxou;

end

plot(a, mamax,'r')

hold on

disp(' ...Overexpression of ComR : done')

for h=1:length(a)

solh = ode23(@OVER_ComS, [t(1) t(end)], [0,a(h),0,0,0,0,0]);

maxou = max(solh.y(6,:));

mamax(h) = maxou;

end

plot(a, mamax,'r')

hold on

disp(' ...Overexpression of ComS : done')

%% Oscillations

disp('Study of oscillations...')

Rrange = max(bR_(t))*[3.25 4 5 7 10];

figure

for ii=1:length((Rrange))

R = Rrange(ii);

sol2 = ode23(@OVER_ComR, [t(1) 600], zeros(1,7));

subplot(2,5,ii)

plot(sol2.x, sol2.y(3,:),'-r')

title('ComS*(t)')

hold on

subplot(2,5,5+ii)

plot(sol2.x, sol2.y(6,:),'-r')

title('ComX(t)')

hold on

end

disp(' ...done')

%% LMG18311

disp('Simulating LMG18311 strain...')

function result = LMG18311(t,y)

dComSdt = bS_(t) + VmaxS*((y(5))/(KS+y(5))) - rout*y(1) - mu_(t)*y(1);

dComSextdt = rout*y(1) - rin*y(2) - mu_(t)*y(2) - dComSext*y(2);

dComSstardt = rin*y(2) - mu_(t)*y(3) - c_RS*(y(3)*y(4))^n - dComSstar*y(3);

dComRdt = (1/10)*bR_(t) + (1/10)*(A/(sigma*sqrt(2*pi)))*exp(-0.5*((t-centre)/sigma)^2) - mu_(t)*y(4) - c_RS*(y(3)*y(4))^n - dComR*y(4);

dComRSdt = (1/2)*c_RS*(y(3)*y(4))^n - mu_(t)*y(5) - vmaxRSZ*(((y(5)*y(7)))/(KRSZ + (y(5)*y(7)))) - dComRS*y(5);

dComXdt = bX_(t) + VmaxX*((y(5))/(KX+y(5))) - mu_(t)*y(6) - dX*y(6);

dComZdt = vmaxZ*((y(6)^nZ)/(KZ^nZ + y(6)^nZ)) - mu_(t)*y(7) -vmaxRSZ*(((y(5)*y(7)))/(KRSZ + (y(5)*y(7)))) - dComZ*y(7);

result = [dComSdt; dComSextdt; dComSstardt; dComRdt; dComRSdt; dComXdt; dComZdt];

end

% Null initial condition

sol2 = ode23(@LMG18311, [t(1) 410], zeros(1,7));

figure

subplot(2,4,1)

plot(sol2.x,X_(sol2.x),'-g')

title('Growth [cells/mL]')

subplot(2,4,2)

plot(sol2.x, sol2.y(1,:),'-r')

hold on

title('ComS(t)')

subplot(2,4,3)

plot(sol2.x, sol2.y(2,:),'-r')

title('ComSext(t)')

hold on

subplot(2,4,4)

plot(sol2.x, sol2.y(3,:),'-r')

title('ComS*(t)')

hold on

subplot(2,4,5)

plot(sol2.x, sol2.y(4,:),'-r')

title('ComR(t)')

hold on

subplot(2,4,6)

plot(sol2.x, sol2.y(5,:),'-r')

title('ComRS(t)')

hold on

subplot(2,4,7)

plot(sol2.x, sol2.y(6,:),'-r')

title('ComX(t)')

hold on

subplot(2,4,8)

plot(sol2.x, sol2.y(7,:),'-r')

title('ComZ(t)')

hold on

% Non-null initial condition : ComSext(t0)=500

sol2 = ode23(@LMG18311, [t(1) 410], [0,500,0,0,0,0,0]);

subplot(2,4,2)

plot(sol2.x, sol2.y(1,:),'-g')

hold on

title('ComS(t)')

subplot(2,4,3)

plot(sol2.x, sol2.y(2,:),'-g')

title('ComSext(t)')

hold on

subplot(2,4,4)

plot(sol2.x, sol2.y(3,:),'-g')

title('ComS*(t)')

hold on

subplot(2,4,5)

plot(sol2.x, sol2.y(4,:),'-g')

title('ComR(t)')

hold on

subplot(2,4,6)

plot(sol2.x, sol2.y(5,:),'-g')

title('ComRS(t)')

hold on

subplot(2,4,7)

plot(sol2.x, sol2.y(6,:),'-g')

title('ComX(t)')

hold on

subplot(2,4,8)

plot(sol2.x, sol2.y(7,:),'-g')

title('ComZ(t)')

hold on

disp(' ...done')

%% Induction factors on ComR

vectorr = [1 2 3 4 10 20 50 100];

L = length(vectorr);

RR = max(bR_(t));

disp('Induction of comR...')

function result = system_ComR_1(t,y)

dComSdt = bS_(t) + VmaxS*((y(5))/(KS+y(5))) - rout*y(1) - mu_(t)*y(1);

dComSextdt = rout*y(1) - rin*y(2) - mu_(t)*y(2) - dComSext*y(2);

dComSstardt = rin*y(2) - mu_(t)*y(3) - c_RS*(y(3)*y(4))^n - dComSstar*y(3);

dComRdt = RR*1 + (A/(sigma*sqrt(2*pi)))*exp(-0.5*((t-centre)/sigma)^2) - mu_(t)*y(4) - c_RS*(y(3)*y(4))^n - dComR*y(4);

dComRSdt = (1/2)*c_RS*(y(3)*y(4))^n - mu_(t)*y(5) - vmaxRSZ*(((y(5)*y(7)))/(KRSZ + (y(5)*y(7)))) - dComRS*y(5);

dComXdt = bX_(t) + VmaxX*((y(5))/(KX+y(5))) - mu_(t)*y(6) - dX*y(6);

dComZdt = vmaxZ*((y(6)^nZ)/(KZ^nZ + y(6)^nZ)) - mu_(t)*y(7) -vmaxRSZ*(((y(5)*y(7)))/(KRSZ + (y(5)*y(7)))) - dComZ*y(7);

result = [dComSdt; dComSextdt; dComSstardt; dComRdt; dComRSdt; dComXdt; dComZdt];

end

function result = system_ComR_2(t,y)

dComSdt = bS_(t) + VmaxS*((y(5))/(KS+y(5))) - rout*y(1) - mu_(t)*y(1);

dComSextdt = rout*y(1) - rin*y(2) - mu_(t)*y(2) - dComSext*y(2);

dComSstardt = rin*y(2) - mu_(t)*y(3) - c_RS*(y(3)*y(4))^n - dComSstar*y(3);

dComRdt = RR*2 + (A/(sigma*sqrt(2*pi)))*exp(-0.5*((t-centre)/sigma)^2) - mu_(t)*y(4) - c_RS*(y(3)*y(4))^n - dComR*y(4);

dComRSdt = (1/2)*c_RS*(y(3)*y(4))^n - mu_(t)*y(5) - vmaxRSZ*(((y(5)*y(7)))/(KRSZ + (y(5)*y(7)))) - dComRS*y(5);

dComXdt = bX_(t) + VmaxX*((y(5))/(KX+y(5))) - mu_(t)*y(6) - dX*y(6);

dComZdt = vmaxZ*((y(6)^nZ)/(KZ^nZ + y(6)^nZ)) - mu_(t)*y(7) -vmaxRSZ*(((y(5)*y(7)))/(KRSZ + (y(5)*y(7)))) - dComZ*y(7);

result = [dComSdt; dComSextdt; dComSstardt; dComRdt; dComRSdt; dComXdt; dComZdt];

end

function result = system_ComR_3(t,y)

dComSdt = bS_(t) + VmaxS*((y(5))/(KS+y(5))) - rout*y(1) - mu_(t)*y(1);

dComSextdt = rout*y(1) - rin*y(2) - mu_(t)*y(2) - dComSext*y(2);

dComSstardt = rin*y(2) - mu_(t)*y(3) - c_RS*(y(3)*y(4))^n - dComSstar*y(3);

dComRdt = RR*3 + (A/(sigma*sqrt(2*pi)))*exp(-0.5*((t-centre)/sigma)^2) - mu_(t)*y(4) - c_RS*(y(3)*y(4))^n - dComR*y(4);

dComRSdt = (1/2)*c_RS*(y(3)*y(4))^n - mu_(t)*y(5) - vmaxRSZ*(((y(5)*y(7)))/(KRSZ + (y(5)*y(7)))) - dComRS*y(5);

dComXdt = bX_(t) + VmaxX*((y(5))/(KX+y(5))) - mu_(t)*y(6) - dX*y(6);

dComZdt = vmaxZ*((y(6)^nZ)/(KZ^nZ + y(6)^nZ)) - mu_(t)*y(7) -vmaxRSZ*(((y(5)*y(7)))/(KRSZ + (y(5)*y(7)))) - dComZ*y(7);

result = [dComSdt; dComSextdt; dComSstardt; dComRdt; dComRSdt; dComXdt; dComZdt];

end

function result = system_ComR_4(t,y)

dComSdt = bS_(t) + VmaxS*((y(5))/(KS+y(5))) - rout*y(1) - mu_(t)*y(1);

dComSextdt = rout*y(1) - rin*y(2) - mu_(t)*y(2) - dComSext*y(2);

dComSstardt = rin*y(2) - mu_(t)*y(3) - c_RS*(y(3)*y(4))^n - dComSstar*y(3);

dComRdt = RR*4 + (A/(sigma*sqrt(2*pi)))*exp(-0.5*((t-centre)/sigma)^2) - mu_(t)*y(4) - c_RS*(y(3)*y(4))^n - dComR*y(4);

dComRSdt = (1/2)*c_RS*(y(3)*y(4))^n - mu_(t)*y(5) - vmaxRSZ*(((y(5)*y(7)))/(KRSZ + (y(5)*y(7)))) - dComRS*y(5);

dComXdt = bX_(t) + VmaxX*((y(5))/(KX+y(5))) - mu_(t)*y(6) - dX*y(6);

dComZdt = vmaxZ*((y(6)^nZ)/(KZ^nZ + y(6)^nZ)) - mu_(t)*y(7) -vmaxRSZ*(((y(5)*y(7)))/(KRSZ + (y(5)*y(7)))) - dComZ*y(7);

result = [dComSdt; dComSextdt; dComSstardt; dComRdt; dComRSdt; dComXdt; dComZdt];

end

function result = system_ComR_10(t,y)

dComSdt = bS_(t) + VmaxS*((y(5))/(KS+y(5))) - rout*y(1) - mu_(t)*y(1);

dComSextdt = rout*y(1) - rin*y(2) - mu_(t)*y(2) - dComSext*y(2);

dComSstardt = rin*y(2) - mu_(t)*y(3) - c_RS*(y(3)*y(4))^n - dComSstar*y(3);

dComRdt = RR*10 + (A/(sigma*sqrt(2*pi)))*exp(-0.5*((t-centre)/sigma)^2) - mu_(t)*y(4) - c_RS*(y(3)*y(4))^n - dComR*y(4);

dComRSdt = (1/2)*c_RS*(y(3)*y(4))^n - mu_(t)*y(5) - vmaxRSZ*(((y(5)*y(7)))/(KRSZ + (y(5)*y(7)))) - dComRS*y(5);

dComXdt = bX_(t) + VmaxX*((y(5))/(KX+y(5))) - mu_(t)*y(6) - dX*y(6);

dComZdt = vmaxZ*((y(6)^nZ)/(KZ^nZ + y(6)^nZ)) - mu_(t)*y(7) -vmaxRSZ*(((y(5)*y(7)))/(KRSZ + (y(5)*y(7)))) - dComZ*y(7);

result = [dComSdt; dComSextdt; dComSstardt; dComRdt; dComRSdt; dComXdt; dComZdt];

end

function result = system_ComR_20(t,y)

dComSdt = bS_(t) + VmaxS*((y(5))/(KS+y(5))) - rout*y(1) - mu_(t)*y(1);

dComSextdt = rout*y(1) - rin*y(2) - mu_(t)*y(2) - dComSext*y(2);

dComSstardt = rin*y(2) - mu_(t)*y(3) - c_RS*(y(3)*y(4))^n - dComSstar*y(3);

dComRdt = RR*20 + (A/(sigma*sqrt(2*pi)))*exp(-0.5*((t-centre)/sigma)^2) - mu_(t)*y(4) - c_RS*(y(3)*y(4))^n - dComR*y(4);

dComRSdt = (1/2)*c_RS*(y(3)*y(4))^n - mu_(t)*y(5) - vmaxRSZ*(((y(5)*y(7)))/(KRSZ + (y(5)*y(7)))) - dComRS*y(5);

dComXdt = bX_(t) + VmaxX*((y(5))/(KX+y(5))) - mu_(t)*y(6) - dX*y(6);

dComZdt = vmaxZ*((y(6)^nZ)/(KZ^nZ + y(6)^nZ)) - mu_(t)*y(7) -vmaxRSZ*(((y(5)*y(7)))/(KRSZ + (y(5)*y(7)))) - dComZ*y(7);

result = [dComSdt; dComSextdt; dComSstardt; dComRdt; dComRSdt; dComXdt; dComZdt];

end

function result = system_ComR_50(t,y)

dComSdt = bS_(t) + VmaxS*((y(5))/(KS+y(5))) - rout*y(1) - mu_(t)*y(1);

dComSextdt = rout*y(1) - rin*y(2) - mu_(t)*y(2) - dComSext*y(2);

dComSstardt = rin*y(2) - mu_(t)*y(3) - c_RS*(y(3)*y(4))^n - dComSstar*y(3);

dComRdt = RR*50 + (A/(sigma*sqrt(2*pi)))*exp(-0.5*((t-centre)/sigma)^2) - mu_(t)*y(4) - c_RS*(y(3)*y(4))^n - dComR*y(4);

dComRSdt = (1/2)*c_RS*(y(3)*y(4))^n - mu_(t)*y(5) - vmaxRSZ*(((y(5)*y(7)))/(KRSZ + (y(5)*y(7)))) - dComRS*y(5);

dComXdt = bX_(t) + VmaxX*((y(5))/(KX+y(5))) - mu_(t)*y(6) - dX*y(6);

dComZdt = vmaxZ*((y(6)^nZ)/(KZ^nZ + y(6)^nZ)) - mu_(t)*y(7) -vmaxRSZ*(((y(5)*y(7)))/(KRSZ + (y(5)*y(7)))) - dComZ*y(7);

result = [dComSdt; dComSextdt; dComSstardt; dComRdt; dComRSdt; dComXdt; dComZdt];

end

function result = system_ComR_100(t,y)

dComSdt = bS_(t) + VmaxS*((y(5))/(KS+y(5))) - rout*y(1) - mu_(t)*y(1);

dComSextdt = rout*y(1) - rin*y(2) - mu_(t)*y(2) - dComSext*y(2);

dComSstardt = rin*y(2) - mu_(t)*y(3) - c_RS*(y(3)*y(4))^n - dComSstar*y(3);

dComRdt = RR*100 + (A/(sigma*sqrt(2*pi)))*exp(-0.5*((t-centre)/sigma)^2) - mu_(t)*y(4) - c_RS*(y(3)*y(4))^n - dComR*y(4);

dComRSdt = (1/2)*c_RS*(y(3)*y(4))^n - mu_(t)*y(5) - vmaxRSZ*(((y(5)*y(7)))/(KRSZ + (y(5)*y(7)))) - dComRS*y(5);

dComXdt = bX_(t) + VmaxX*((y(5))/(KX+y(5))) - mu_(t)*y(6) - dX*y(6);

dComZdt = vmaxZ*((y(6)^nZ)/(KZ^nZ + y(6)^nZ)) - mu_(t)*y(7) -vmaxRSZ*(((y(5)*y(7)))/(KRSZ + (y(5)*y(7)))) - dComZ*y(7);

result = [dComSdt; dComSextdt; dComSstardt; dComRdt; dComRSdt; dComXdt; dComZdt];

end

mamax = zeros(1, L);

sol = ode23(@system_ComR_1, [t(1) t(end)], zeros(1,7));

maxou = max(sol.y(6,:));

mamax(1) = maxou;

sol = ode23(@system_ComR_2, [t(1) t(end)], zeros(1,7));

maxou = max(sol.y(6,:));

mamax(2) = maxou;

sol = ode23(@system_ComR_3, [t(1) t(end)], zeros(1,7));

maxou = max(sol.y(6,:));

mamax(3) = maxou;

sol = ode23(@system_ComR_4, [t(1) t(end)], zeros(1,7));

maxou = max(sol.y(6,:));

mamax(4) = maxou;

sol = ode23(@system_ComR_10, [t(1) t(end)], zeros(1,7));

maxou = max(sol.y(6,:));

mamax(5) = maxou;

sol = ode23(@system_ComR_20, [t(1) t(end)], zeros(1,7));

maxou = max(sol.y(6,:));

mamax(6) = maxou;

sol = ode23(@system_ComR_50, [t(1) t(end)], zeros(1,7));

maxou = max(sol.y(6,:));

mamax(7) = maxou;

sol = ode23(@system_ComR_100, [t(1) t(end)], zeros(1,7));

maxou = max(sol.y(6,:));

mamax(8) = maxou;

figure

plot(vectorr, mamax,'*r-')

hold on

title('Effet of ComR production on ComX')

disp(' ...done')

%% Induction factors on ComS

SS = max(bS_(t));

disp('Induction of comS...')

function result = system_ComS_1(t,y)

dComSdt = SS*1 + VmaxS*((y(5))/(KS+y(5))) - rout*y(1) - mu_(t)*y(1);

dComSextdt = rout*y(1) - rin*y(2) - mu_(t)*y(2) - dComSext*y(2);

dComSstardt = rin*y(2) - mu_(t)*y(3) - c_RS*(y(3)*y(4))^n - dComSstar*y(3);

dComRdt = bR_(t) + (A/(sigma*sqrt(2*pi)))*exp(-0.5*((t-centre)/sigma)^2) - mu_(t)*y(4) - c_RS*(y(3)*y(4))^n - dComR*y(4);

dComRSdt = (1/2)*c_RS*(y(3)*y(4))^n - mu_(t)*y(5) - vmaxRSZ*(((y(5)*y(7)))/(KRSZ + (y(5)*y(7)))) - dComRS*y(5);

dComXdt = bX_(t) + VmaxX*((y(5))/(KX+y(5))) - mu_(t)*y(6) - dX*y(6);

dComZdt = vmaxZ*((y(6)^nZ)/(KZ^nZ + y(6)^nZ)) - mu_(t)*y(7) -vmaxRSZ*(((y(5)*y(7)))/(KRSZ + (y(5)*y(7)))) - dComZ*y(7);

result = [dComSdt; dComSextdt; dComSstardt; dComRdt; dComRSdt; dComXdt; dComZdt];

end

function result = system_ComS_2(t,y)

dComSdt = SS*2 + VmaxS*((y(5))/(KS+y(5))) - rout*y(1) - mu_(t)*y(1);

dComSextdt = rout*y(1) - rin*y(2) - mu_(t)*y(2) - dComSext*y(2);

dComSstardt = rin*y(2) - mu_(t)*y(3) - c_RS*(y(3)*y(4))^n - dComSstar*y(3);

dComRdt = bR_(t) + (A/(sigma*sqrt(2*pi)))*exp(-0.5*((t-centre)/sigma)^2) - mu_(t)*y(4) - c_RS*(y(3)*y(4))^n - dComR*y(4);

dComRSdt = (1/2)*c_RS*(y(3)*y(4))^n - mu_(t)*y(5) - vmaxRSZ*(((y(5)*y(7)))/(KRSZ + (y(5)*y(7)))) - dComRS*y(5);

dComXdt = bX_(t) + VmaxX*((y(5))/(KX+y(5))) - mu_(t)*y(6) - dX*y(6);

dComZdt = vmaxZ*((y(6)^nZ)/(KZ^nZ + y(6)^nZ)) - mu_(t)*y(7) -vmaxRSZ*(((y(5)*y(7)))/(KRSZ + (y(5)*y(7)))) - dComZ*y(7);

result = [dComSdt; dComSextdt; dComSstardt; dComRdt; dComRSdt; dComXdt; dComZdt];

end

function result = system_ComS_3(t,y)

dComSdt = SS*3 + VmaxS*((y(5))/(KS+y(5))) - rout*y(1) - mu_(t)*y(1);

dComSextdt = rout*y(1) - rin*y(2) - mu_(t)*y(2) - dComSext*y(2);

dComSstardt = rin*y(2) - mu_(t)*y(3) - c_RS*(y(3)*y(4))^n - dComSstar*y(3);

dComRdt = bR_(t) + (A/(sigma*sqrt(2*pi)))*exp(-0.5*((t-centre)/sigma)^2) - mu_(t)*y(4) - c_RS*(y(3)*y(4))^n - dComR*y(4);

dComRSdt = (1/2)*c_RS*(y(3)*y(4))^n - mu_(t)*y(5) - vmaxRSZ*(((y(5)*y(7)))/(KRSZ + (y(5)*y(7)))) - dComRS*y(5);

dComXdt = bX_(t) + VmaxX*((y(5))/(KX+y(5))) - mu_(t)*y(6) - dX*y(6);

dComZdt = vmaxZ*((y(6)^nZ)/(KZ^nZ + y(6)^nZ)) - mu_(t)*y(7) -vmaxRSZ*(((y(5)*y(7)))/(KRSZ + (y(5)*y(7)))) - dComZ*y(7);

result = [dComSdt; dComSextdt; dComSstardt; dComRdt; dComRSdt; dComXdt; dComZdt];

end

function result = system_ComS_4(t,y)

dComSdt = SS*4 + VmaxS*((y(5))/(KS+y(5))) - rout*y(1) - mu_(t)*y(1);

dComSextdt = rout*y(1) - rin*y(2) - mu_(t)*y(2) - dComSext*y(2);

dComSstardt = rin*y(2) - mu_(t)*y(3) - c_RS*(y(3)*y(4))^n - dComSstar*y(3);

dComRdt = bR_(t) + (A/(sigma*sqrt(2*pi)))*exp(-0.5*((t-centre)/sigma)^2) - mu_(t)*y(4) - c_RS*(y(3)*y(4))^n - dComR*y(4);

dComRSdt = (1/2)*c_RS*(y(3)*y(4))^n - mu_(t)*y(5) - vmaxRSZ*(((y(5)*y(7)))/(KRSZ + (y(5)*y(7)))) - dComRS*y(5);

dComXdt = bX_(t) + VmaxX*((y(5))/(KX+y(5))) - mu_(t)*y(6) - dX*y(6);

dComZdt = vmaxZ*((y(6)^nZ)/(KZ^nZ + y(6)^nZ)) - mu_(t)*y(7) -vmaxRSZ*(((y(5)*y(7)))/(KRSZ + (y(5)*y(7)))) - dComZ*y(7);

result = [dComSdt; dComSextdt; dComSstardt; dComRdt; dComRSdt; dComXdt; dComZdt];

end

function result = system_ComS_10(t,y)

dComSdt = SS*10 + VmaxS*((y(5))/(KS+y(5))) - rout*y(1) - mu_(t)*y(1);

dComSextdt = rout*y(1) - rin*y(2) - mu_(t)*y(2) - dComSext*y(2);

dComSstardt = rin*y(2) - mu_(t)*y(3) - c_RS*(y(3)*y(4))^n - dComSstar*y(3);

dComRdt = bR_(t) + (A/(sigma*sqrt(2*pi)))*exp(-0.5*((t-centre)/sigma)^2) - mu_(t)*y(4) - c_RS*(y(3)*y(4))^n - dComR*y(4);

dComRSdt = (1/2)*c_RS*(y(3)*y(4))^n - mu_(t)*y(5) - vmaxRSZ*(((y(5)*y(7)))/(KRSZ + (y(5)*y(7)))) - dComRS*y(5);

dComXdt = bX_(t) + VmaxX*((y(5))/(KX+y(5))) - mu_(t)*y(6) - dX*y(6);

dComZdt = vmaxZ*((y(6)^nZ)/(KZ^nZ + y(6)^nZ)) - mu_(t)*y(7) -vmaxRSZ*(((y(5)*y(7)))/(KRSZ + (y(5)*y(7)))) - dComZ*y(7);

result = [dComSdt; dComSextdt; dComSstardt; dComRdt; dComRSdt; dComXdt; dComZdt];

end

function result = system_ComS_20(t,y)

dComSdt = SS*20 + VmaxS*((y(5))/(KS+y(5))) - rout*y(1) - mu_(t)*y(1);

dComSextdt = rout*y(1) - rin*y(2) - mu_(t)*y(2) - dComSext*y(2);

dComSstardt = rin*y(2) - mu_(t)*y(3) - c_RS*(y(3)*y(4))^n - dComSstar*y(3);

dComRdt = bR_(t) + (A/(sigma*sqrt(2*pi)))*exp(-0.5*((t-centre)/sigma)^2) - mu_(t)*y(4) - c_RS*(y(3)*y(4))^n - dComR*y(4);

dComRSdt = (1/2)*c_RS*(y(3)*y(4))^n - mu_(t)*y(5) - vmaxRSZ*(((y(5)*y(7)))/(KRSZ + (y(5)*y(7)))) - dComRS*y(5);

dComXdt = bX_(t) + VmaxX*((y(5))/(KX+y(5))) - mu_(t)*y(6) - dX*y(6);

dComZdt = vmaxZ*((y(6)^nZ)/(KZ^nZ + y(6)^nZ)) - mu_(t)*y(7) -vmaxRSZ*(((y(5)*y(7)))/(KRSZ + (y(5)*y(7)))) - dComZ*y(7);

result = [dComSdt; dComSextdt; dComSstardt; dComRdt; dComRSdt; dComXdt; dComZdt];

end

function result = system_ComS_50(t,y)

dComSdt = SS*50 + VmaxS*((y(5))/(KS+y(5))) - rout*y(1) - mu_(t)*y(1);

dComSextdt = rout*y(1) - rin*y(2) - mu_(t)*y(2) - dComSext*y(2);

dComSstardt = rin*y(2) - mu_(t)*y(3) - c_RS*(y(3)*y(4))^n - dComSstar*y(3);

dComRdt = bR_(t) + (A/(sigma*sqrt(2*pi)))*exp(-0.5*((t-centre)/sigma)^2) - mu_(t)*y(4) - c_RS*(y(3)*y(4))^n - dComR*y(4);

dComRSdt = (1/2)*c_RS*(y(3)*y(4))^n - mu_(t)*y(5) - vmaxRSZ*(((y(5)*y(7)))/(KRSZ + (y(5)*y(7)))) - dComRS*y(5);

dComXdt = bX_(t) + VmaxX*((y(5))/(KX+y(5))) - mu_(t)*y(6) - dX*y(6);

dComZdt = vmaxZ*((y(6)^nZ)/(KZ^nZ + y(6)^nZ)) - mu_(t)*y(7) -vmaxRSZ*(((y(5)*y(7)))/(KRSZ + (y(5)*y(7)))) - dComZ*y(7);

result = [dComSdt; dComSextdt; dComSstardt; dComRdt; dComRSdt; dComXdt; dComZdt];

end

function result = system_ComS_100(t,y)

dComSdt = SS*100 + VmaxS*((y(5))/(KS+y(5))) - rout*y(1) - mu_(t)*y(1);

dComSextdt = rout*y(1) - rin*y(2) - mu_(t)*y(2) - dComSext*y(2);

dComSstardt = rin*y(2) - mu_(t)*y(3) - c_RS*(y(3)*y(4))^n - dComSstar*y(3);

dComRdt = bR_(t) + (A/(sigma*sqrt(2*pi)))*exp(-0.5*((t-centre)/sigma)^2) - mu_(t)*y(4) - c_RS*(y(3)*y(4))^n - dComR*y(4);

dComRSdt = (1/2)*c_RS*(y(3)*y(4))^n - mu_(t)*y(5) - vmaxRSZ*(((y(5)*y(7)))/(KRSZ + (y(5)*y(7)))) - dComRS*y(5);

dComXdt = bX_(t) + VmaxX*((y(5))/(KX+y(5))) - mu_(t)*y(6) - dX*y(6);

dComZdt = vmaxZ*((y(6)^nZ)/(KZ^nZ + y(6)^nZ)) - mu_(t)*y(7) -vmaxRSZ*(((y(5)*y(7)))/(KRSZ + (y(5)*y(7)))) - dComZ*y(7);

result = [dComSdt; dComSextdt; dComSstardt; dComRdt; dComRSdt; dComXdt; dComZdt];

end

sol = ode23(@system_ComS_1, [t(1) t(end)], zeros(1,7));

maxou = max(sol.y(6,:));

mamax(1) = maxou;

sol = ode23(@system_ComS_2, [t(1) t(end)], zeros(1,7));

maxou = max(sol.y(6,:));

mamax(2) = maxou;

sol = ode23(@system_ComS_3, [t(1) t(end)], zeros(1,7));

maxou = max(sol.y(6,:));

mamax(3) = maxou;

sol = ode23(@system_ComS_4, [t(1) t(end)], zeros(1,7));

maxou = max(sol.y(6,:));

mamax(4) = maxou;

sol = ode23(@system_ComS_10, [t(1) t(end)], zeros(1,7));

maxou = max(sol.y(6,:));

mamax(5) = maxou;

sol = ode23(@system_ComS_20, [t(1) t(end)], zeros(1,7));

maxou = max(sol.y(6,:));

mamax(6) = maxou;

sol = ode23(@system_ComS_50, [t(1) t(end)], zeros(1,7));

maxou = max(sol.y(6,:));

mamax(7) = maxou;

sol = ode23(@system_ComS_100, [t(1) t(end)], zeros(1,7));

maxou = max(sol.y(6,:));

mamax(8) = maxou;

figure

plot(vectorr(:,1:end), mamax,'*r-')

hold on

title('Effet of ComS production on ComX')

disp(' ...done')

%% SENSIBILITY ANALYSIS

disp('Sensitivity analysis...')

foldchange = [0.1 0.25 0.5 0.6 0.7 0.8 0.9 1 1.5 2 2.5 3 3.5 4 10];

% change here the parameter to be tested

param_range1 = rout*foldchange;

maximaComX = zeros(size(foldchange));

for ii=1:length((foldchange))

rout = param_range1(ii);

sol2 = ode23(@system_S1, [t(1) t(end)], zeros(1,7));

maxou = max(sol2.y(6,:));

maximaComX(ii) = maxou;

if foldchange(ii) == 1

currentvalue = maxou;

end

end

figure

plot(foldchange,maximaComX,'-r',1,currentvalue,'b*')

disp(' ...done')

%% Different timings for ComS adjunction

disp('Simulating different timings for ComS adding...')

add_value = 150; % Number of added ComS molecules

integ_time = 360; % Integration time

sol2 = ode23(@KO_ComS, [0 integ_time], [0,add_value,0,0,0,0,0]);

figure

plot(sol2.x,sol2.y(6,:),'r')

hold on

add_time = 30:30:330; % time points for adjunction of ComS

for index=1:length(add_time)

sol_1 = ode23(@KO_ComS, [0 add_time(index)], zeros(7,1));

sol_2 = ode23(@KO_ComS, [add_time(index) integ_time], [sol_1.y(1,end); sol_1.y(2,end)+ add_value; sol_1.y(3,end); sol_1.y(4,end); sol_1.y(5,end); sol_1.y(6,end); sol_1.y(7,end);]);

plot([sol_1.x,sol_2.x], [sol_1.y(6,:),sol_2.y(6,:)],'-r')

hold on

end

disp(' ...done')

%% Mimicking different peptide affinities

disp('Mimicking different peptide affinities...')

a = [0 1 2 5 10 20 50 100 200 500 1000 2000];

cRS_test = [1 0.5 0.1 0.1/5 0.1/10 0.1/20 0.1/28 0.1/35 0.1/100 0.1/500 0.1/1000 0.1/2000 0.1/5000 0.1/10000 0.1/20000 0];

figure

for ii=1:length(cRS_test)

c_RS = cRS_test(ii);

mamax_X = zeros(1, length(a));

for h=1:length(a)

solh = ode23(@KO_ComS, [t(1) t(end)], [0,a(h),0,0,0,0,0]);

maxou_X = max(solh.y(6,:));

mamax_X(h) = maxou_X;

end

plot(a, mamax_X,'r')

hold on

end

disp(' ...done')

disp('Hallelujah :-)')

end
