# Supplementary material for: Modeling of the ComRS Signaling Pathway Reveals the Limiting Factors Controlling Competence in Streptococcus thermophilus
Source: Front Microbiol. 2015 Dec 22;6:1413. doi: 10.3389/fmicb.2015.01413 (PMC4686606; doi:10.3389/fmicb.2015.01413)
Supplement: Supplementary file 3 [file DataSheet3.pdf]

Supplementary Material

Modeling of the ComRS signaling pathway reveals the limiting factors controlling competence in *Streptococcus thermophilus*

Laurie Haustenne[1], Georges Bastin[2], Pascal Hols[1$] and Laetitia Fontaine[1*$]

[1] Biochimie, Biophysique et Génétique des Microorganismes, Institut des Sciences de la Vie, Université catholique de Louvain, B-1348 Louvain-la-Neuve, Belgium.

[2] Center for Systems Engineering and Applied Mechanics, ICTEAM, Université catholique de Louvain, B-1348 Louvain-la-Neuve, Belgium.

***Correspondence:** Laetitia Fontaine; Biochimie, Biophysique et Génétique des Microorganismes; Institut des Sciences de la Vie, Université catholique de Louvain, 4-5 Place Croix du Sud (L7.07.06), 1348, Louvain-la-Neuve. laetitia.fontaine@uclouvain.be

[$] Both authors contributed equally to this work

1. Supplementary Data

1.1. Supplementary Data Sheets

Supplementary Data Sheet 1. | MATLAB file. This file contains the MATLAB code (as a text file) used for model building, validation, and outputs. Please copy-paste all the text in a new MATLAB file named ‘ODE_model.m’. Supplementary Data Sheet 2 is required to run the model.

Supplementary Data Sheet 2. | Experimental data sheet. This xlsx file contains all the experimental data needed (growth and luciferase curves) to run the model. Please rename it as “Experimental_data.xlsx” and copy this file in the same folder as the MATLAB file ‘ODE_model.m’.

2. Supplementary Figures and Tables

2.1. Supplementary Tables

Supplementary Table 2. | Bacterial strains and plasmids used in this study.

Strain	Genotype	Characteristic(s)[a]	Source or reference
Streptococcus thermophilus			
LMD-9	Wild type		ATCC[b]
CB001	LMD-9 (*blpD-blpX*)::P_{comX}-*luxAB*		(Fontaine et al., 2010a)
LF125	CB001 *comX*::P32-*cat*	Cm^R	(Boutry et al., 2013)
LF118	CB001 *comS*::P32-*cat*	Cm^R	(Fontaine et al., 2013)
TIL1391	CB001 *comS*::*spec*	Spc^R	(Gardan et al., 2013)
LH001	CB001 *dprA*::P32-*cat*	Cm^R	This study
LF121	LMD-9 (*blpD-blpX*)::P_{comS}-*luxAB*		This study
LF122	LF121 *comX*::P32-*cat*	Cm^R	(Boutry et al., 2013)
LF134	LF121 *comS*::P32-*cat*	Cm^R	(Fontaine et al., 2013)
LF135	LF121 *comR*::P32-*cat*	Cm^R	(Fontaine et al., 2013)
LH002	LF121 *dprA*::P32-*cat*	Cm^R	This study
LF123	LMD-9 (*blpD-blpX*)::P_{comR}-*luxAB*		(Boutry et al., 2013)
LMG18311	Wild type		BBCM LMG[c]
CB009	LMG18311 (*blpU-blpX*)::P_{comX}-*luxAB*		This study
LF146	LMG18311 (*blpU-blpX*)::P_{comR}-*luxAB-spec*	Spc^R	This study
Escherichia coli			
EC1000	MC1000 containing a copy of the *repA* gene of pWV01 in its chromosome	Km^R $RepA^+$	(Law et al., 1995)

Plasmid	Description	Characteristic(s)[a]	Source or reference

pNZ5319	pACYC184 derivative containing the *lox66*-P32-*cat*-*lox71* cassette	Cm^R Em^R	(Lambert et al., 2007)
pGIUD0855cat	pUC18 derivative used to assess natural transformation rates of *S. thermophilus* LMG18311 derivative strains	Ap^R Cm^R	(Fontaine et al., 2010b)
pR412	plasmid used to amplify the spectinomycin resistance cassette P_{spec}-*spec*	Spc^R	(Martin et al., 2000)
pGICB004	pG+host9 derivative used to integrate the *luxAB* reporter genes by double cross-over at the *blp* locus of *S. thermophilus*LMG18311	Em^R Ts	(Fleuchot et al., 2011)
pGICB001	pGICB004 derivative used to integrate the P_{comX}-*luxAB* transcriptional fusion by double cross-over at the *blp* locus of *S. thermophilus* LMG18311	Em^R Ts	(Fontaine et al., 2010a)
pGILFspec	pGICB004 derivative containing the spectinomycin resistance cassette P_{spec}-*spec* downstream of *luxAB*	Em^R Spc^R Ts	This study
pGILFspec::P_{comR}	pGILFspec derivative used to introduce a P_{comR}-*luxAB*-*spec* transcriptional fusion at the *blp* locus of of *S. thermophilus* LMG18311	Em^R Spc^R Ts	This study
pMG36eT	pMG36e derivative used for the constitutive expression of genes under the control of the P32 promoter	Em^R	Fontaine and Hols, 2008
pMGcomRstrep	pMG36eT derivative used for the constitutive expression of $comR_{\mathrm{LMD9}}$::streptagII fusion under the control of the P32 promoter	Em^R	(Boutry et al., 2013)

[a] Cm^R , Em^R, Spc^R and Ts indicate resistance to chloramphenicol, erythromycin and spectinomycin, and that the plasmid encodes a thermosensitive RepA protein, respectively.
[b] ATCC, American Type Culture Collection, Rockville, MD.
[c] BCCM Belgian Coordinated Collections of Microorganisms, LMG Laboratory of Microbiology and Genetics, Ghent, Belgium.

Supplementary Table 2. | Primers used for plasmid and strain construction.

Primer	Sequence (5 '→ 3')	DNA target[a]	restriction sites[b]	name of constructed strain or plasmid	Source or reference
**Primers used to amplify the *lox66*-P32-*cat*-*lox71* cassette[c]**					
Uplox66	TAAGGAAGATAAATCCCATAAGG	upstream *lox66* (plasmid pNZ5319)			(Fontaine et al., 2010b)
DNlox71	TTCACGTTACTAAAGGGAATGTA	downstream *lox71* (plasmid pNZ5319)			
**Primers used to create overlap PCR products containing the *lox66*-P32-*cat*-*lox71* cassette. The PCR products are then transformed by competence to create mutant strains[c]**					
UpdprA_A	CTCTGTCGGTTACCCAATATTTGCGTGCTC	upstream *dprA*		LH002	This study
UpdprA_B	CCTTATGGGATTTATCTTCCTTATTCAAAGT TATTCATCTAAC				
DNdprA_A	TACATTCCCTTTAGTAACGTGAATCAGAATT TTCATAAAAATC	downstream *dprA*			
DNdprA_B	TTCGGTCCAAAACACGACGAGCTTGCTGAG				
**Primers used for the validation of mutant or reporter strains obtained by natural transformation**					
ChdprA_A	TAAGAGTGCTATTGGTGTTCTCTTGC	upstream *dprA* (strains LH001 and LH002)		LH002	This study
ChdprA_B	TCATGGAATTTCACCTCAATTTCTTGC	downstream *dprA* (strains LH001 and LH002)			
INDISBAC1	TTAATGATAAACCAAGAAGAGTGG	*blpH* (CB009, LF123, LF146)		CB009, LF123, LF146	(Fontaine et al., 2007)
INTDISTIX2	TTATAACCAGTTCTGGCATGACCG	*pepX* (CB009, LF123, LF146)			This study
**Primers used for plasmid construction**					
LoxSpec-Sma-F	AAAACCCGGGATAAGGAAGATAAATCCCAT	P_{spec}-*spec*	*Sma*I	pGILFspec	This study

	AAGGATACCGTTCGTATAATGTATGCTATA CGAAGTTATTCGATACCGTCGACCTCG	cassette (plasmid pR412)			
LoxSpec-Pvu-R	AAATCAGCTGTTCACGTTACTAAAGGGAAT GTAATACCGTTCGTATAGCATACATTATAC GAAGTTATAGCTCGAATTGACGCGGAATGG		*Pvu*II		
JUDPster0316A	GGACTAGTTGCATATTTTTGTTGGATAATCA AG	P_{comR} (*S. thermophilus* LMG18311)	*Spe*I	pGILFspec::PcomR	(Fontaine et al., 2013)
JUDPster0316B	CGGAATTCAAGTTCAAGAGAATCTCCTTTA		*Eco*RI		(Fontaine et al., 2013)
**Primers used to amplify the Cy3-P_{comS} probe**					
Cy3PcomS1A	CAGGAAAATTGGCAGATGGTTTATAG	P_{comS} (*S. thermophilus* LMG18311)			(Fontaine et al., 2013)
pMGSHP16sur2	TTTCTGCAGTTACATTTTGGCATGATGGCTC C				

[a] Refer to the hybridization target of primers. Unless otherwise stated (between brackets), template chromosomal DNA is from *S. thermophilus* LMD-9 WT.
[b] Restriction sites introduced in primers for cloning purposes are underlined.
[c] The sequence in the primer that allows overlapping with the *lox66*-P32-*cat*-*lox71* cassette is in bold type.

2.2. Supplementary Figures

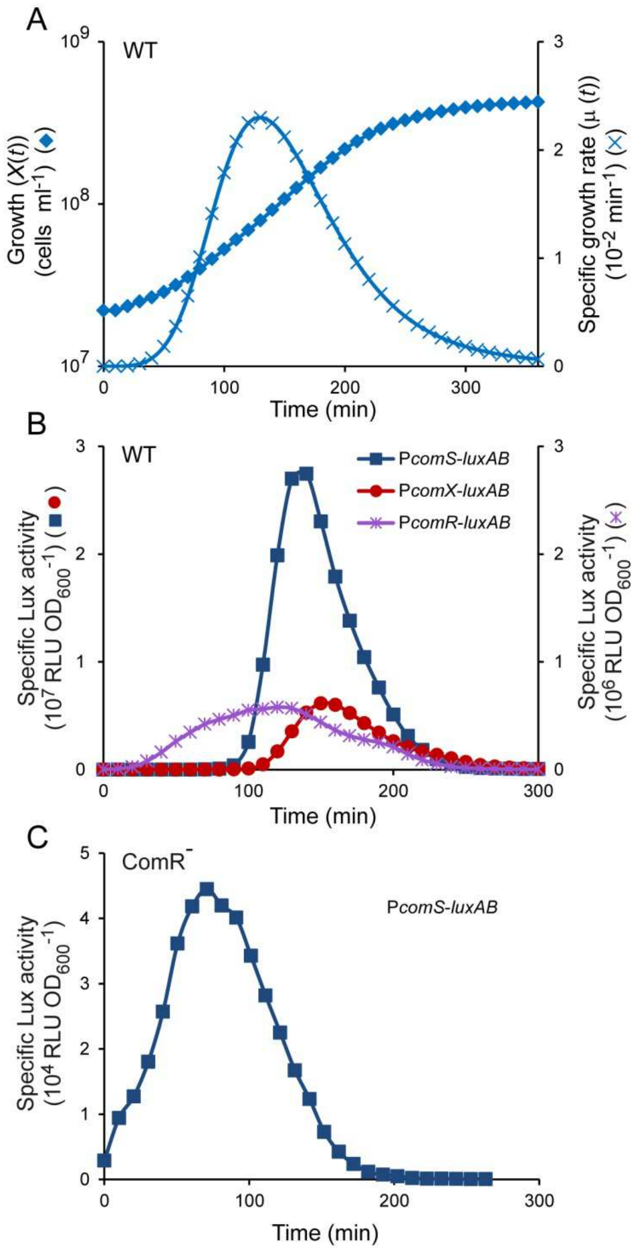

Supplementary Figure S1. | Experimental data used to parametrize the ComRS model. (A) Kinetics of growth ($X(t)$; expressed in cell ml^{-1}; first *y* axis) and specific growth rate ($\mu(t)$; expressed in min^{-1}; second *y* axis) of WT *S. thermophilus* LMD-9. $\mu(t)$ was calculated from $X(t)$ using the equation $\mu(t) = dX(t)/dt \times 1/X(t)$. To calculate $X(t)$, OD_{600} of the P_{comX} (CB001), P_{comS} (LF121) and P_{comR} (LF123) WT reporter strains was measured every 10 min. The mean OD600 values measured at each time (i.e. mean for the 3 reporter strains) were then converted in # cells ml^{-1} by assuming that a OD_{600} unit of 1 = 5 × 10^8 cells ml^{-1} (see DataSheet 2). This conversion was deduced form a plating and CFU (colony forming unit) counting experiment. Culture samples were taken at regular intervals during growth, diluted, plated on M17L, and incubated at 37°C until CFU counting. These curves were used to model growth in system S1. **(B)** Kinetics of specific luciferase activity (RLU OD_{600}^{-1}) during growth of the LMD-9 reporter strains bearing the transcriptional fusion P_{comS}-*luxAB* (LF121), P_{comX}-*luxAB* (CB001) (first *y* axis) and P_{comR}-*luxAB* (LF123) (second *y* axis). These curves were used to replace theoretical time-varying production rates of ComS, ComX and ComR, respectively, in system S2, and to parametrize the modeled activation terms of system S1 (see Materials and Methods). **(C)** Kinetics of specific luciferase activity (RLU OD_{600}^{-1}) of the LMD-9 $ComR^-$ reporter strain encoding a transcriptional P_{comS}-*luxAB* fusion (strain LF135). This curve was used to calculate the theoretical time-varying basal production rate of ComS ($b_S(t)$), ComX ($b_X(t)$) and ComR ($b_R(t)$) in system S1 using equation (14) (see Materials and methods).

All strains were grown in CDML. Each curve represents the average from three independent repeats. Curves of panels A, B and C were adapted from Fontaine et al. (2013).

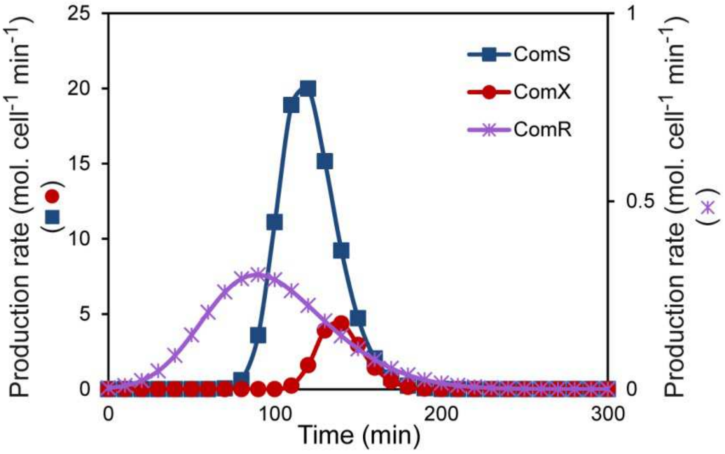

Supplementary Figure S2. ǀ Experimental production rates. Kinetics of experimental production rates (expressed in molecules (mol.) cell^{-1} min^{-1}) of ComS, ComX (first *y* axis), and ComR (second *y* axis). These production rates were deduced from the specific luciferase activities driven from promoters P_{comS}, P_{comX}, and P_{comR}, respectively (see Supplementary Figure S1B) using equation (13). These experimental production rates were used to replace modeled time-varying production rates of ComS, ComX and ComR, respectively, in system S2, and to parametrize the modeled activation terms of system S1 (see Materials and Methods for detailed information).

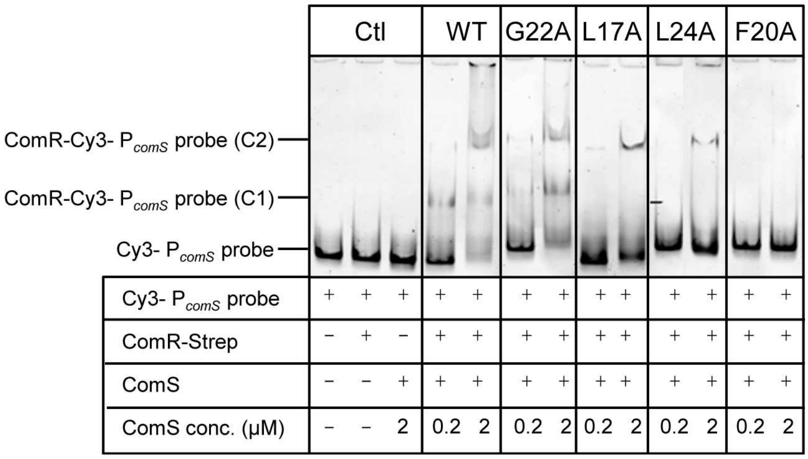

Supplementary Figure S3. ǀ Efficiency of ComR binding to the promoter region of *comS* in presence of different $ComS_{17\text{-}24}$ variants. EMSA experiments showing the binding of purified ComR-Strep to promoter P_{comS}. 150 ng Cy3-P_{comS} probe (encompassing the entire P_{comS} region) was incubated in the presence (+) or absence (-) of purified $ComR_{LMD\text{-}9}$-Strep (4 µM) and $ComS_{17\text{-}24}$ (0.2 and 2 µM) before separation on a TBE gel under non-denaturating conditions. Unmodified (WT) or modified $ComS_{17\text{-}24}$ octapeptide containing one alanine substitution at position 22 (G22A), 17 (L17A), 24 (L24A) and 20 (F20A) of the full-length ComS peptide. These variants (from left to

right) display a decreasing ability to stimulate binding of ComR to P_{comS}. Ctl, negative controls; C1: protein-DNA complex 1; C2: protein-DNA complex 2.

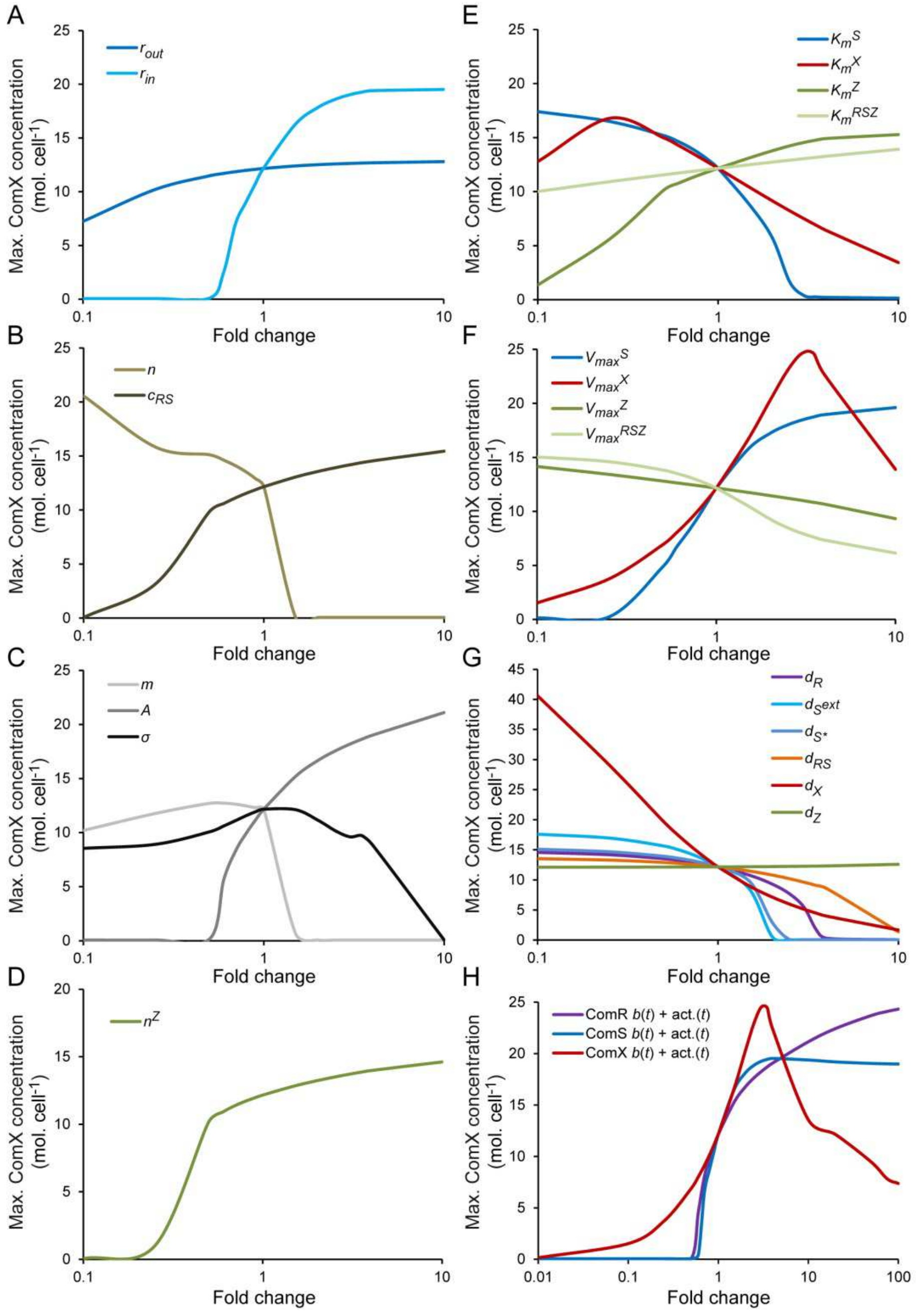

Supplementary Figure S4. | Sensitivity analysis of system S1. Maximum ComX concentration (molecules (mol.) cell^{-1}) computed when varying a given parameter value in system S1 within a fold change interval ranging of 0.1- to 10 times its assigned value (0.01 to 100 in panel H). Parameters were grouped according to their nature or specific role in ODE equations. They describe **(A)** ComS importation and exportation, **(B)** ComRS complexes formation, **(C)** the activation of ComR production, **(D)** the cooperativity of complex formation (hill coefficient), **(E)** the affinity of

interactions (K_m constants), **(F)** the maximum rate of reactions (V_{max}), **(G)** degradation of modeled molecular species, and **(H)** production of ComR, ComS and ComX due to basal production $b(t)$ and activation (act.(t)). Parameters and their assigned value in system S1 are summarized in Table 2.

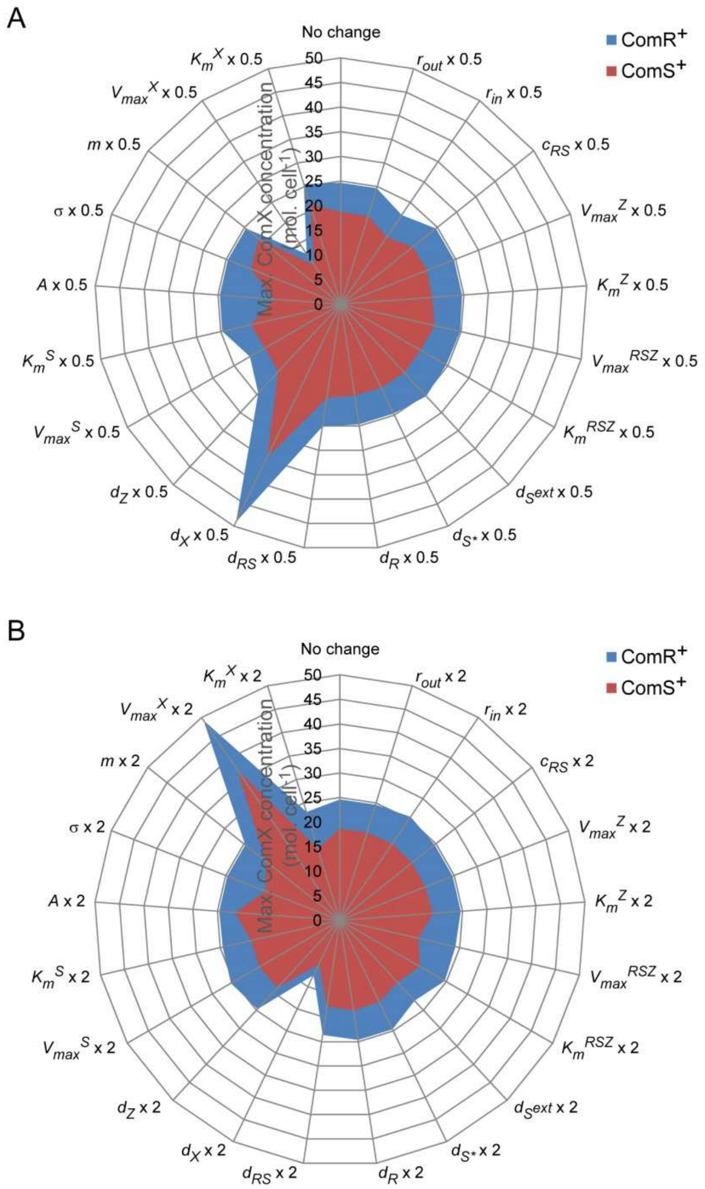

Supplementary Figure S5. | Impact of parameter values of system S1 on the maximum ComX concentration (molecules (mol.) cell^{-1}) computed for the simulated ComR^{+} and ComS^{+} strains. ComR^{+} (blue surface) and ComS^{+} (red surface) strains were simulated by increasing the maximal basal production rate of variables ComR(t) and ComS(t) by a constant inducing factor (IF) of 100. Then, the numerical value of 20 parameters of system S1 was individually varied within a fold change interval ranging from 0.5 to 2 times its assigned value. **(A)** Numerical value of each parameter multiplied by 0.5 (× 0.5). **(B)** Numerical value of each parameter multiplied by 2 (× 2). Parameters and their values in system S1 are listed in **Table 2**. This figure shows that higher ComX concentrations are reached in the ComR^{+} vs. ComS^{+} cells for each modified numerical value.

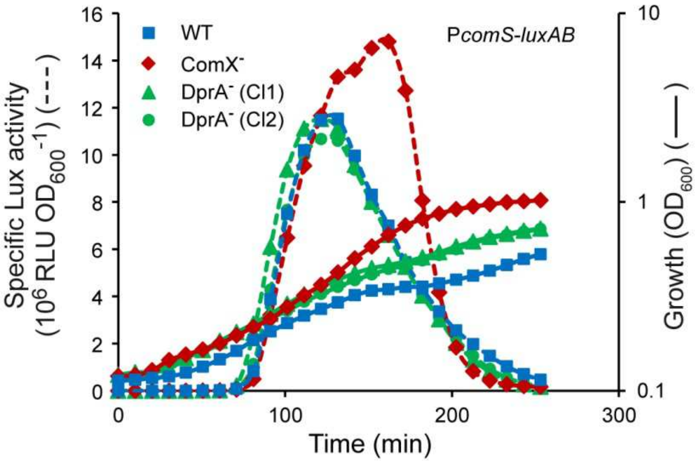

Supplementary Figure S6. | Role of DprA in competence shut-off. Kinetics of specific luciferase activity (first *y* axis; RLU OD_{600}^{-1}) and growth (second *y* axis; OD_{600}) of different derivatives of strain LF121 (carrying the P_{comS}-*luxAB* transcriptional fusion) grown in CDML medium: WT (LF121), $DprA^-$ (LH002, clones Cl1 and Cl2) and $ComX^-$ (LF122).

References

Boutry, C., Delplace, B., Clippe, A., Fontaine, L., and Hols, P. (2013). SOS response activation and competence development are antagonistic mechanisms in *Streptococcus thermophilus*. *J. Bacteriol.* 195, 696-707.

Fleuchot, B., Gitton, C., Guillot, A., Vidic, J., Nicolas, P., Besset, C., et al. (2011). Rgg proteins associated with internalized small hydrophobic peptides: a new quorum-sensing mechanism in streptococci. *Mol. Microbiol.* 80, 1102-1119.

Fontaine, L., Boutry, C., de Frahan, M. H., Delplace, B., Fremaux, C., Horvath, P., et al. (2010a). A novel pheromone quorum-sensing system controls the development of natural competence in *Streptococcus thermophilus* and *Streptococcus salivarius*. *J. Bacteriol.* 192, 1444-1454.

Fontaine, L., Boutry, C., Guedon, E., Guillot, A., Ibrahim, M., Grossiord, B., et al. (2007). Quorum-sensing regulation of the production of Blp bacteriocins in *Streptococcus thermophilus*. *J. Bacteriol.* 189, 7195-7205.

Fontaine, L., Dandoy, D., Boutry, C., Delplace, B., de Frahan, M. H., Fremaux, C., et al. (2010b). Development of a versatile procedure based on natural transformation for marker-free targeted genetic modification in *Streptococcus thermophilus*. *Appl. Environ. Microbiol.* 76, 7870-7877.

Fontaine, L., Goffin, P., Dubout, H., Delplace, B., Baulard, A., Lecat-Guillet, N., et al. (2013). Mechanism of competence activation by the ComRS signalling system in streptococci. *Mol. Microbiol.* 87, 1113-1132.

Gardan, R., Besset, C., Gitton, C., Guillot, A., Fontaine, L., Hols, P., et al. (2013). Extracellular life cycle of ComS, the competence-stimulating peptide of *Streptococcus thermophilus*. *J. Bacteriol.* 195, 1845-1855.

Lambert, J. M., Bongers, R. S., and Kleerebezem, M. (2007). Cre-lox-based system for multiple gene deletions and selectable-marker removal in *Lactobacillus plantarum*. *Appl. Environ. Microbiol.* 73, 1126-1135.

Law, J., Buist, G., Haandrikman, A., Kok, J., Venema, G., and Leenhouts, K. (1995). A system to generate chromosomal mutations in *Lactococcus lactis* which allows fast analysis of targeted genes. *J. Bacteriol.* 177, 7011-7018.

Martin, B., Prudhomme, M., Alloing, G., Granadel, C., and Claverys, J. P. (2000). Cross-regulation of competence pheromone production and export in the early control of transformation in *Streptococcus pneumoniae*. *Mol. Microbiol.* 38, 867-878.
